# Type 3 secretion system induced leukotriene B4 synthesis by leukocytes is actively inhibited by *Yersinia pestis* to evade early immune recognition

**DOI:** 10.1371/journal.ppat.1011280

**Published:** 2024-01-25

**Authors:** Amanda Brady, Katelyn R. Sheneman, Amanda R. Pulsifer, Sarah L. Price, Taylor M. Garrison, Krishna Rao Maddipati, Sobha R. Bodduluri, Jianmin Pan, Nolan L. Boyd, Jing-Juan Zheng, Shesh N. Rai, Jason Hellmann, Bodduluri Haribabu, Silvia M. Uriarte, Matthew B. Lawrenz

**Affiliations:** 1 Department of Microbiology and Immunology, University of Louisville School of Medicine, Louisville, Kentucky, United States of America; 2 Department of Pathology, Lipidomics Core Facility, Wayne State University, Detroit, Michigan, United States of America; 3 Biostatistics and Bioinformatics Facility, Brown Cancer Center, University of Louisville, Louisville, Kentucky, United States of America; 4 Center for Cardiometabolic Science, Christina Lee Brown Environment Institute, Division of Environmental Medicine, University of Louisville School of Medicine, Louisville, Kentucky, United States of America; 5 Department of Pharmacology and Toxicology, University of Louisville School of Medicine, Louisville, Kentucky, United States of America; 6 Deptartment of Oral Immunology & Infectious Diseases, University of Louisville, Louisville, Kentucky, United States of America; 7 Center for Predictive Medicine for Biodefense and Emerging Infectious Diseases, Louisville, Kentucky, United States of America; Tufts University, UNITED STATES

## Abstract

Subverting the host immune response to inhibit inflammation is a key virulence strategy of *Yersinia pestis*. The inflammatory cascade is tightly controlled via the sequential action of lipid and protein mediators of inflammation. Because delayed inflammation is essential for *Y*. *pestis* to cause lethal infection, defining the *Y*. *pestis* mechanisms to manipulate the inflammatory cascade is necessary to understand this pathogen’s virulence. While previous studies have established that *Y*. *pestis* actively inhibits the expression of host proteins that mediate inflammation, there is currently a gap in our understanding of the inflammatory lipid mediator response during plague. Here we used the murine model to define the kinetics of the synthesis of leukotriene B4 (LTB_4_), a pro-inflammatory lipid chemoattractant and immune cell activator, within the lungs during pneumonic plague. Furthermore, we demonstrated that exogenous administration of LTB_4_ prior to infection limited bacterial proliferation, suggesting that the absence of LTB_4_ synthesis during plague contributes to *Y*. *pestis* immune evasion. Using primary leukocytes from mice and humans further revealed that *Y*. *pestis* actively inhibits the synthesis of LTB_4_. Finally, using *Y*. *pestis* mutants in the Ysc type 3 secretion system (T3SS) and *Yersinia* outer protein (Yop) effectors, we demonstrate that leukocytes recognize the T3SS to initiate the rapid synthesis of LTB_4_. However, several Yop effectors secreted through the T3SS effectively inhibit this host response. Together, these data demonstrate that *Y*. *pestis* actively inhibits the synthesis of the inflammatory lipid LTB_4_ contributing to the delay in the inflammatory cascade required for rapid recruitment of leukocytes to sites of infection.

## Author summary

*Yersinia pestis* targets the host’s innate immune response to inhibit inflammation. Because the generation of a non-inflammatory environment is required for infection, defining the mechanisms used by *Y*. *pestis* to block inflammation is key to understanding its virulence. Lipid mediators are potent signaling molecules required for timely initiation of host inflammation. While previous studies have demonstrated that *Y*. *pestis* inhibits inflammation, there is a gap in our understanding of the inflammatory lipid mediator response during plague. Here we show that *Y*. *pestis* inhibits the production of one of these critical lipid mediators, leukotriene B4, by host immune cells. Furthermore, we identify both the signals that induce LTB_4_ production by leukocytes and the mechanisms used by *Y*. *pestis* to inhibit this process. Together, these data represent the first comprehensive analysis of inflammatory lipids produced during pneumonic plague and improve our current understanding of how *Y*. *pestis* manipulates the host immune response to generate a permissive non-inflammatory environment required to colonize the mammalian host.

## Introduction

*Yersinia pestis* causes the human disease known as the plague. Although typically characterized as a disease of our past, in the aftermath of the 3^rd^ plague pandemic, *Y*. *pestis* became endemic in rodent populations in several countries, increasing the potential for spillover into human populations through contact with infected animals and fleas [1–3]. Human plague manifests in three forms: bubonic, septicemic, or pneumonic plague. Bubonic plague resulting from flea transmission arises when bacteria colonize and replicate within lymph nodes. Septicemic plague results when *Y*. *pestis* gains access to the bloodstream, either directly from a flea bite or via dissemination from an infected lymph node, and results in uncontrolled bacterial replication and sepsis. Finally, secondary pneumonic plague, wherein *Y*. *pestis* disseminates to the lungs via the blood, results in a pneumonia that can promote direct person-to-person transmission via aerosols. While treatable with antibiotics, if left untreated, all forms of plague are associated with high mortality rates, and the probability of successful treatment decreases the longer initiation of treatment is delayed post-exposure [3–6]. Regardless of the route of infection, one of the key virulence determinants for *Y*. *pestis* to colonize the host is the Ysc type 3 secretion system (T3SS) encoded on the pCD1 plasmid [5,7]. This secretion system allows direct translocation of bacterial effector proteins, called Yops, into host cells [5,8,9]. The Yop effectors target specific host factors to disrupt normal host cell signaling pathways and functions [10–15]. Because the T3SS and Yops are required for mammalian but not flea infection, the expression of the genes encoding these virulence factors are differentially expressed within these two hosts [5,8,16,17]. The primary signal leading to T3SS and Yop expression is a shift in temperature from that of the flea vector (<28°C) to that of the mammalian host (>30°C). During mammalian infection, *Y*. *pestis* primarily targets neutrophils and macrophages for T3SS-mediated injection of the Yop effectors [18–20]. The outcomes of Yop injection into these cells include inhibition of phagocytosis, reactive oxygen species (ROS) synthesis, degranulation by neutrophils, and inflammatory cytokine and chemokine release required to recruit circulating neutrophils to infection sites [21–26]. Importantly, previous work suggests that inhibition of neutrophil influx and establishing a non-inflammatory environment is crucial for *Y*. *pestis* virulence [27,28]. Therefore, defining the molecular mechanisms used by *Y*. *pestis* to subvert the host immune response is fundamental to understanding the pathogenesis of this organism. Moreover, defining the host mechanisms targeted by *Y*. *pestis* to inhibit inflammation can also provide novel insights into how the host responds to bacterial pathogens to control infection.

**Fig 1:**
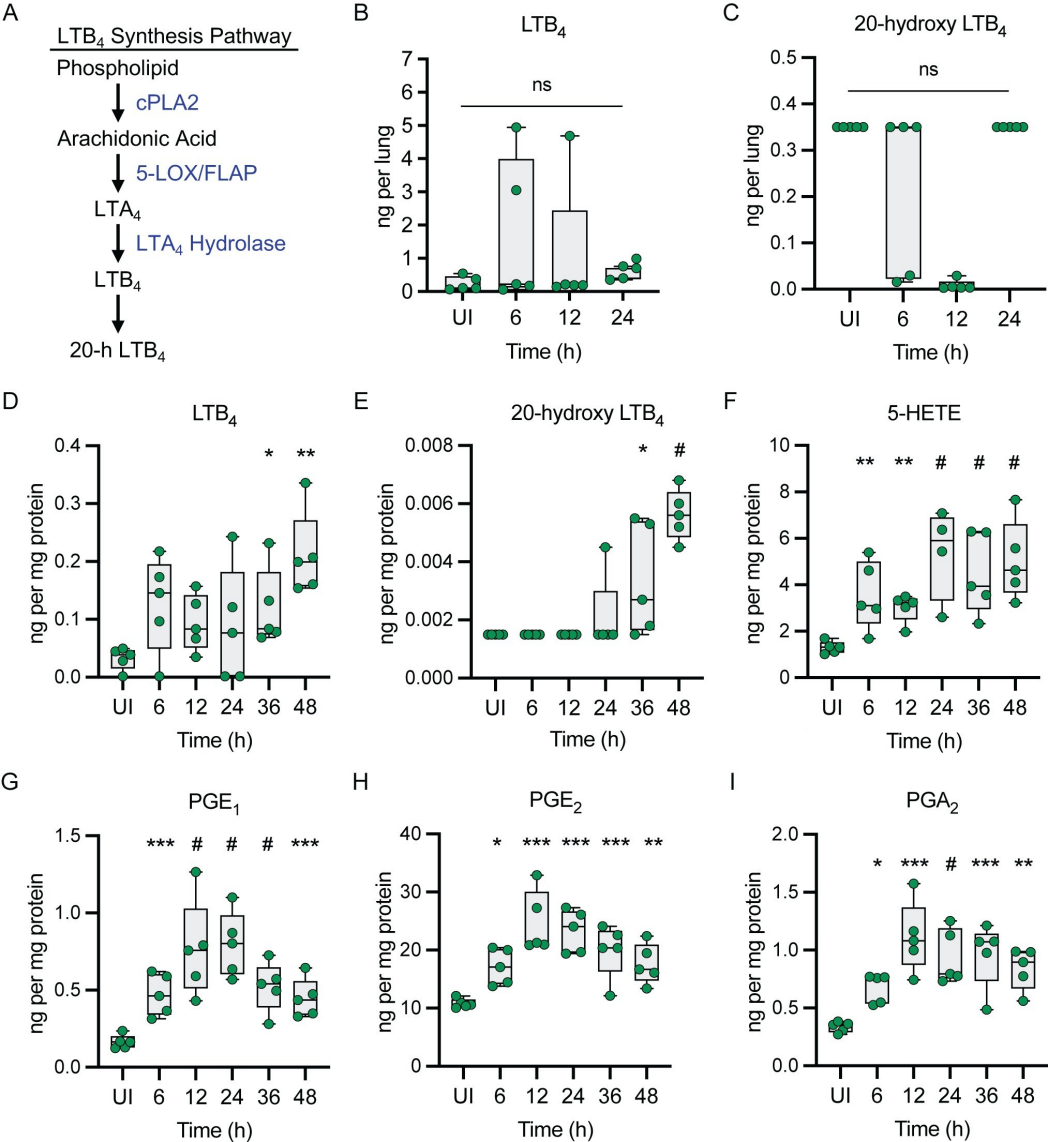
LTB_4_ synthesis is blunted during pneumonic plague. (A) The LTB_4_ synthesis pathway. (B-I) C56BL/6J mice were infected with 10 x the LD_50_ *Y*. *pestis* KIM5+ and lungs were harvested at the indicated times (n = 5) to measure host lipids by LC-MS. UI = samples from uninfected animals. (B) LTB_4_ concentrations. (C) 20-hydroxy LTB_4_ concentrations. (D) LTB_4_ concentrations. (E) 20-hydroxy LTB_4_ concentrations. (F) 5-HETE concentrations. (G) PGE_1_ concentrations. (H) PGE_2_ concentrations. (I) PGA_2_ concentrations. Each symbol represents an individual mouse and the box plot represents the median of the group ± the range. Changes in lipid concentrations were compared to the UI sample using (B-C) One-way ANOVA with Dunnett’s *post hoc* test or (D-I) the LIMMA—Moderated t-test. ns = not significant, * = p ≤ 0.05, ** = p ≤ 0.01, *** = p ≤ 0.001, # = p ≤ 0.0001.

A cascade of events tightly regulates inflammation to ensure rapid responses to control infection and effective immune resolution after clearance of pathogens to limit tissue damage [29,30]. This inflammatory cascade is initiated by synthesizing potent lipid mediators and is sustained and amplified by the subsequent production of protein mediators [31,32]. Polyunsaturated fatty acid (PUFA)-derived lipid mediators are potent modulators of the innate and adaptive immune responses [30,33]. Of these, the eicosanoids, including the leukotrienes and the prostaglandins, are key regulators of the inflammatory cascade during infection [31,32]. Leukotriene B4 (LTB_4_) is rapidly synthesized from arachidonic acid upon activation of 5-lipoxygenase (5-LOX), cytosolic phospholipase A_2_ (cPLA2), 5-LOX activating protein (FLAP), and LTA_4_ hydrolase (Fig 1A) [34]. Upon synthesis and release, LTB_4_ is recognized by the high affinity BLT1 receptor on immune cells to promote chemotaxis and initiate the inflammatory cascade leading to production of pro-inflammatory cytokines and chemokines [31,32,35–39]. Together these inflammatory mediators promote the recruitment of circulating leukocytes to infected tissue [32]. Importantly, because of its critical role in initiating the inflammatory cascade, disruption in the timely production of LTB_4_ can slow the subsequent downstream release of cytokines and chemokines and the ability of the host to mount a rapid inflammatory response required to control infection.

Despite active proliferation of *Y*. *pestis* within the lungs in the mouse model, there appears to be an absence of pro-inflammatory cytokines, chemokines, and neutrophil influx for the first 36 hours of primary pneumonic plague [11–15]. This phenotype dramatically differs from pulmonary infection with attenuated mutants of *Y*. *pestis* lacking the T3SS or Yop effectors or by other pulmonary pathogens, such as *Klebsiella pneumoniae*, which induce significant inflammation within 24 hours of bacterial exposure [11–15]. Surprisingly, despite the importance of lipid mediators in initiating the inflammatory cascade, the role of inflammatory lipids during plague has not been previously investigated. However, using human peripheral blood neutrophils, Pulsifer et al. previously demonstrated that *Y*. *pestis* can actively inhibit the synthesis of LTB_4_ *in vitro* in a T3SS/Yop-dependent manner, suggesting that LTB_4_ synthesis may be inhibited during plague [26]. In this study, we expand on these observations by investigating LTB_4_ synthesis by the mammalian host in response to *Y*. *pestis*. Using the murine model of plague, we demonstrate dysregulation in the production of LTB_4_ by *Y*. *pestis* and provide the lipidomic profile of other host inflammatory lipids during the initial 48 h of pneumonic plague. We further show that exogenous treatment with LTB_4_ inhibits bacterial proliferation in the murine model. Using *Y*. *pestis* mutants, we also discovered that leukocyte interactions with the *Y*. *pestis* T3SS triggers LTB_4_ synthesis, but synthesis is inhibited by multiple Yop effectors secreted via the same T3SS. Together, these data suggest that modulation in the production of host inflammatory lipids is an additional virulence mechanism used by *Y*. *pestis* to inhibit the rapid recruitment of immune cells needed to control infection.

## Results

### LTB_4_ synthesis is delayed during pneumonic plague

Based on our previous observations that *Y*. *pestis* inhibits LTB_4_ synthesis by human neutrophils [26], we sought to determine if LTB_4_ was synthesized during infection using the murine model of pneumonic plague. C57BL/6J mice were intranasally infected with *Y*. *pestis* KIM5+ and LTB_4_ was measured in the lungs during the non-inflammatory stage of disease (6, 12, and 24 h post-infection). We did not observe a statistically significant increase in LTB_4_ synthesis at any time point, with only 3 of the 15 samples having elevated LTB_4_ concentrations over the entire 24 h period (Fig 1B). Moreover, we did not observe a significant increase in 20-hydroxy LTB_4_ (Fig 1C), which is the direct degradation product of LTB_4_ (Fig 1A). To confirm these results, LTB_4_ was measured in the lungs from a second independent group of C57BL/6J mice, this time expanding the analysis to include the pro-inflammatory stage of disease (36 and 48 h post-infection). Again, we did not observe statistically significant increases in LTB_4_ or 20-hydroxy LTB_4_ during the first 24 h of infection (Fig 1D and 1E). However, by 36 h post-infection, both lipids were statistically elevated compared to uninfected samples (p ≤ 0.05). Moreover, we observed a significant increase in 5-HETE as early as 6 h post-infection (Fig 1F; p ≤ 0.01), which can result if 5-LOX does not complete the synthesis of LTA_4_ from arachidonic acid [40,41]. While the synthesis of LTB_4_ appears absent during the first 24 h of infection, the synthesis of other inflammatory lipids increased during the same period, including the prostaglandins, which are another group of eicosanoids whose synthesis is regulated via the cyclooxygenase pathway (Fig 1G–1I). Globally, we observed significant changes in the synthesis of 63 lipids during pneumonic plague, including lipids generally considered to be pro-inflammatory (18 lipids), anti-inflammatory (41 lipids), or pro-resolving (4 lipids) (S1 Table) [42,43]. Together these data indicate LTB_4_ synthesis is delayed during pneumonic plague.

### BLT1^-/-^ mice are not more susceptible to pneumonic plague than C57BL/6J mice

**Fig 2:**
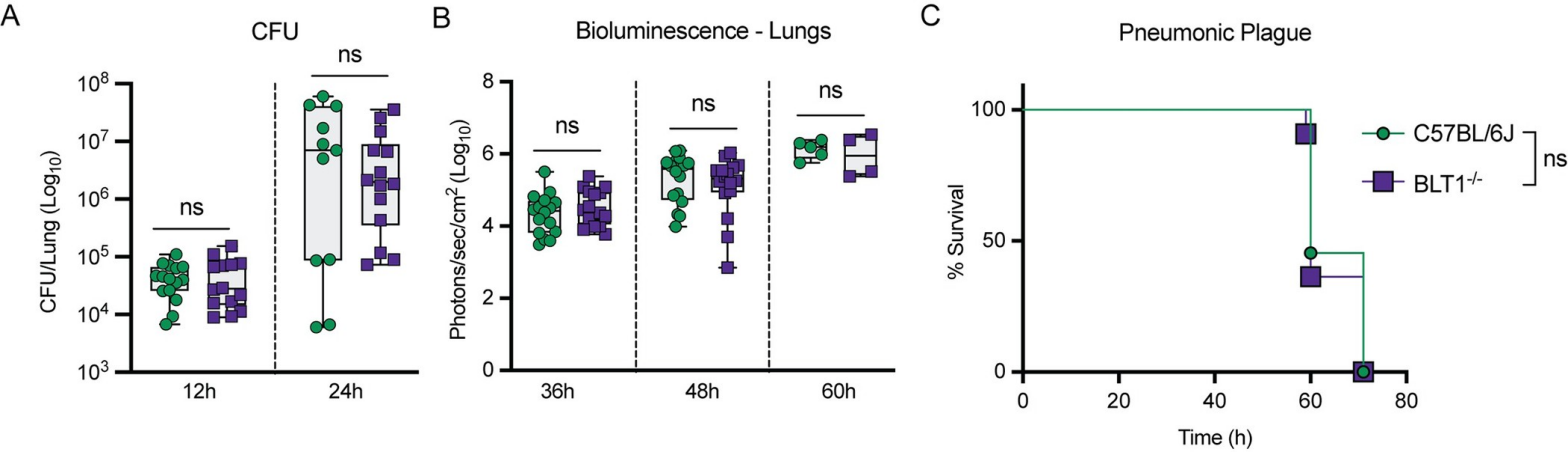
BLT1^-/-^ mice are not more susceptible to pneumonic plague than C57BL/6J mice. (A) C57BL/6J (green circles) or BLT1^-/-^ (purple squares) mice were infected intranasally with 10 x the LD_50_ of *Y*. *pestis* KIM5+ and lungs were harvested at 12 and 24 h post-infection. Bacterial proliferation within the lungs was determined by CFU enumeration. Each symbol represents an indivdual mouse and the box plot represents the median of the group ± the range. Combined data from two independent experiments. (B-C) C57BL/6J (green circles) or BLT1^-/-^ (purple squares) mice were infected intranasally with 10x the LD_50_ of *Y*. *pestis* CO92 LUX_p*cysZK*_. (B) Bacterial proliferation in the lungs as a function of bioluminescence. Each symbol represents an indivdual mouse and the box plot represents the median of the group ± the range. Combined data from two independent experiments. (C) Survival curves of mice from B (n = 15). For A and B, T-test with Mann-Whitney’s *post hoc* test indicated no statistically significant (ns) differences between C57BL/6J and BLT1^-/-^ groups. For C, Log-Rank anlysis revealed no statistically signficant (ns) differences in surival between the two groups.

LTB_4_ is recognized by the high-affinity G-protein coupled receptor BLT1, which is expressed primarily by innate and adaptive immune cells [38,44]. LTB_4_-BLT1 engagement leads to host inflammatory immune responses such as chemotaxis, cytokine release, phagocytosis, and ROS production that contribute to the clearance of pathogens [35,45]. Mice deficient in the expression of BLT1 cannot effectively respond to LTB_4_ signaling and are generally more susceptible to infection [39,46,47]. Because we did not observe LTB_4_ synthesis during the early stages of pneumonic plague, we hypothesized that BLT1^-/-^ mice would not be more susceptible to *Y*. *pestis* infection. To test this hypothesis, we intranasally infected C57BL/6J and BLT1^-/-^ mice with *Y*. *pestis* KIM5+ and measured bacterial numbers in the lungs at 12 and 24 h post-infection. Bacterial numbers were not significantly higher in BLT1^-/-^ mice than C57BL/6J mice (Fig 2A). Furthermore, independent experiments with a *Y*. *pestis* strain with a luciferase bioreporter (*Y*. *pestis* CO92 Lux_*pcysZK*_), which allows for monitoring bacterial proliferation via optical imaging and host survival in the same group [48], showed no significant differences between the two mouse lines in bacterial proliferation at later time points or in the mean-time to death (Fig 2B and 2C). These data indicate that the loss of LTB_4_-BLT1 signaling in BLT1^-/-^ mice does not impact the infectivity of *Y*. *pestis*, further supporting that LTB_4_ synthesis and signaling is disrupted during pneumonic plague.

### Exogenous LTB_4_ treatment limits *Y*. *pestis* proliferation *in vivo*

**Fig 3:**
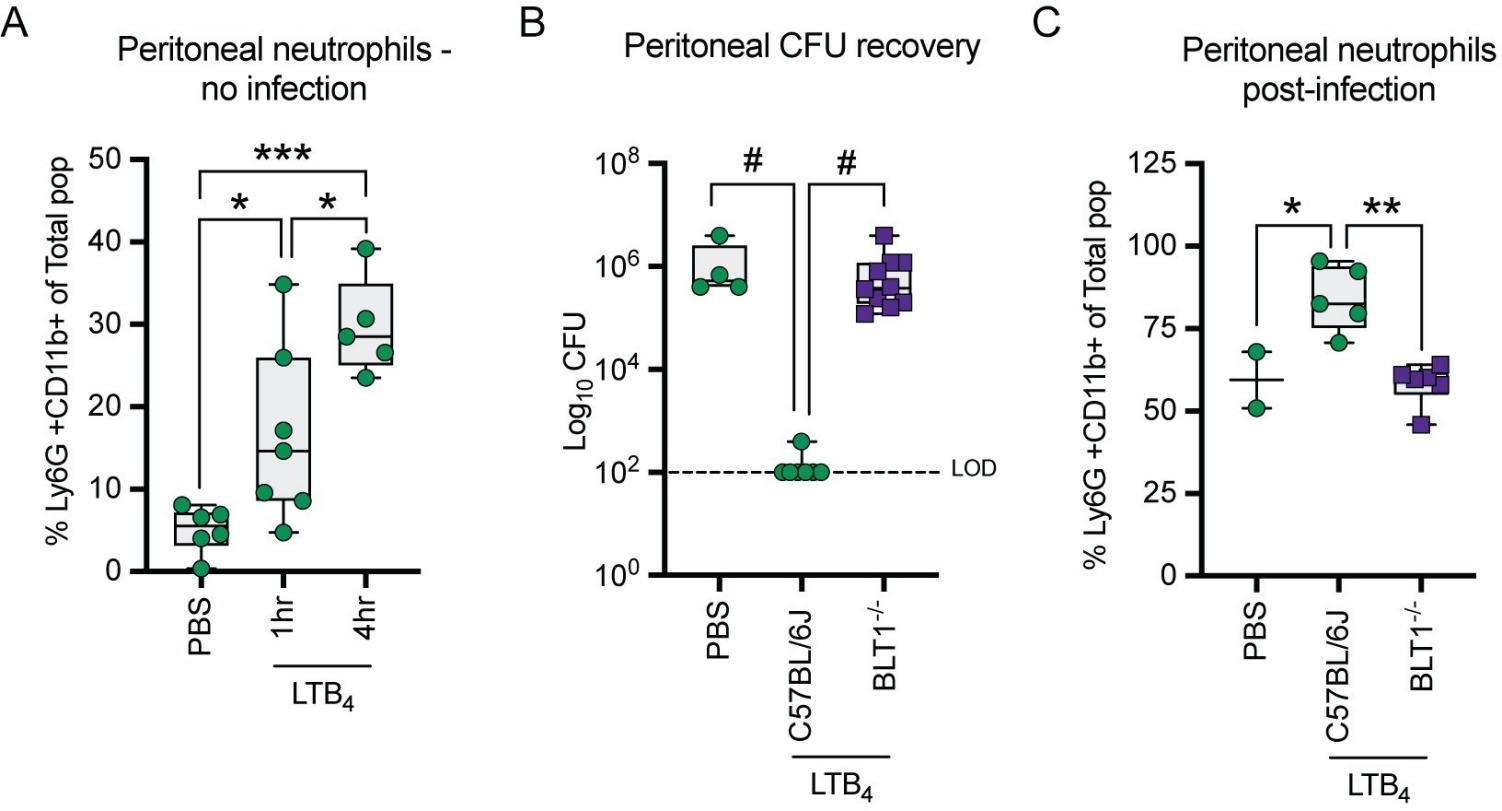
LTB_4_ treatment improves host killing of *Y*. *pestis*. (A) C57BL/6J mice were administered 1 x DPBS (PBS) or 10 nmol LTB_4_ intraperitoneally and changes in neutrophil populations (Ly6G+CD11b+) were measured at 1 or 4 h post-treatment. (B) C57BL/6J (green circles) or BLT1^-/-^ (purple squares) mice administered DPBS or 10 nmol LTB_4_ were infected 1 h later with 10^5^ CFU of *Y*. *pestis* KIM5+ and bacterial numbers in the peritoneal cavities were enumerated 3 h post-inoculation. LOD = Limit of detection. (C) Neutrophil populations from a subset of animals from B. Each symbol represents an indivdual mouse and the box plot represents the median of the group ± the range. Combined data from three independent experiments. One-way ANOVA with Tukey’s *post hoc* test compared to each condition. * = p ≤ 0.05, ** = p ≤ 0.01, *** = p ≤ 0.001, # = p ≤ 0.0001.

Because LTB_4_ synthesis and signaling appears to be disrupted during infection, we next asked if exogenous administration of LTB_4_ could alter infection. To test this hypothesis, we used a previously described peritoneal model that allows for accurate administration of LTB_4_ and easy recovery of both elicited leukocytes and bacteria via lavage [49]. As described previously, intraperitoneal administration of LTB_4_ resulted in an increase in the neutrophil population within the peritoneal cavity in C57BL/6J mice as early as 1 h post-administration (Figs 3A and S1) [49]. When challenged intraperitoneally with *Y*. *pestis* KIM5+ after LTB_4_ administration, we observed a significant decrease in the number of viable bacteria recovered from LTB_4_-treated animals, approaching the limit of detection, as compared to PBS-treated animals 3 h post-infection (Fig 3B; p ≤ 0.0001). Neutrophil numbers also remained significantly elevated in the LTB_4_-treated animals at 3 h post-infection compared to PBS-treated animals (Fig 3C; p ≤ 0.05). Moreover, bacterial clearance was dependent on LTB_4_ signaling, as LTB_4_ treatment of BLT1^-/-^ mice did not alter bacterial or neutrophil numbers at 3 h post-infection compared to PBS-treated C57BL/6J mice (Fig 3B and 3C). Together, these data indicate that LTB_4_-mediated recruitment and activation of leukocytes can improve the host response to *Y*. *pestis*.

### Neutrophils do not synthesize LTB_4_ in response to *Y*. *pestis*

**Fig 4:**
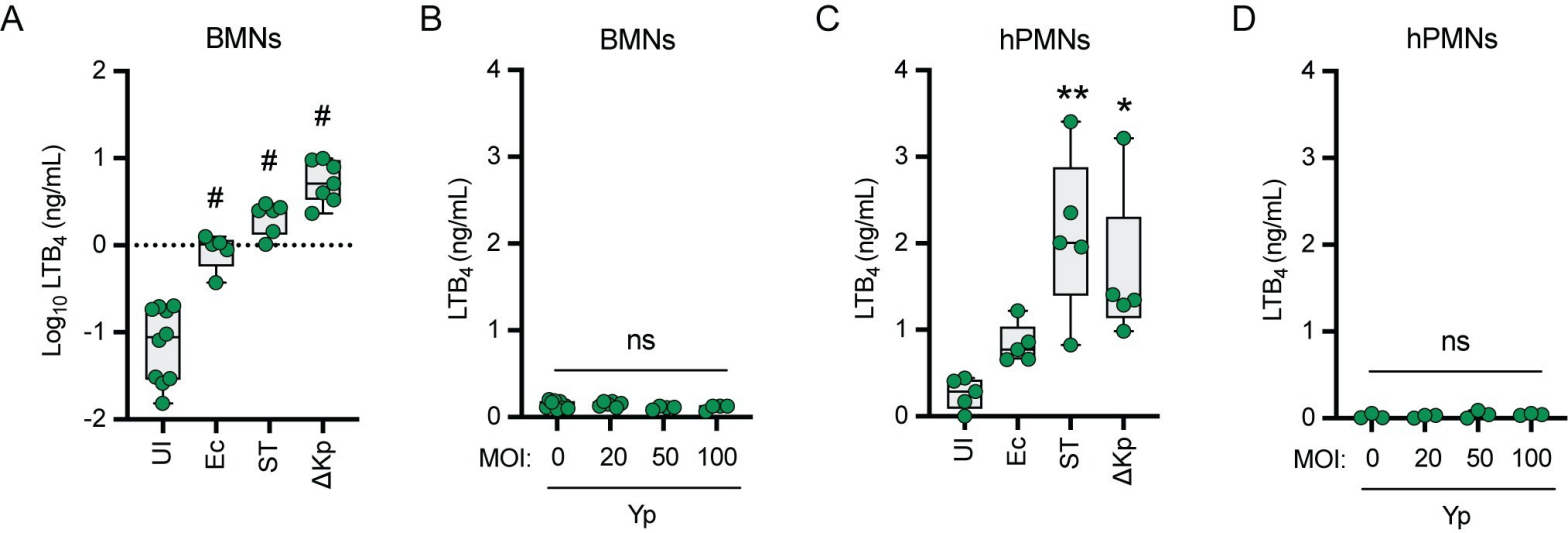
Neutrophils do not synthesize LTB_4_ in response to *Y*. *pestis*. (A-B) Murine (BMNs) or (C-D) human (hPMNs) neutrophils were infected with *E*. *coli* DH5α (Ec), *S*. *enterica* Typhimurium LT2 (ST), or *K*. *pneumoniae manC* (ΔKp) at an MOI of 20, or with *Y*. *pestis* (Yp) at increasing MOIs. LTB_4_ was measured from supernatants 1 h post infection by ELISA. Each symbol represents an independent biological infection and the box plot represents the median of the group ± the range. UI or 0 = uninfected. One-way ANOVA with Dunnett’s *post hoc* test compared to uninfected. * = p ≤ 0.05, ** = p ≤ 0.01, # = p ≤ 0.0001.

Because neutrophils are robust sources of LTB_4_ [35], are the primary cells with which *Y*. *pestis* interacts during the first 24 h of pneumonic plague [19], and *Y*. *pestis* inhibits LTB_4_ synthesis by human neutrophils [26], we next sought to determine if the LTB_4_ response by neutrophils differed between *Y*. *pestis* and other bacteria. When bone marrow-derived neutrophils (BMNs) from C57BL/6J mice were stimulated with *E*. *coli*, *S*. *enterica* Typhimurium, or a *K*. *pneumoniae manC* mutant (unable to synthesize a capsule), LTB_4_ synthesis was significantly induced within 1 h of infection (Fig 4A; p ≤ 0.0001). However, infection with *Y*. *pestis* did not elicit LTB_4_ synthesis, even when the MOI was increased to 100 bacteria per neutrophil (Fig 4B). Similar phenotypes were observed during infection of human peripheral blood neutrophils (hPMNs), recapitulating our previously published data for *Y*. *pestis* (Fig 4C and 4D) [26]. Importantly, the absence of LTB_4_ synthesis did not appear to be due to *Y*. *pestis* induced cell death, as no significant changes in cell permeability or cytotoxicity were observed during *Y*. *pestis* infections at an MOI of 20 when compared to uninfected neutrophils (S2A and S2B Fig). Even at an MOI of 100, while cell permeability appeared slightly elevated in *Y*. *pestis*-infected murine neutrophils compared to uninfected cells (S2C Fig; 9% vs. 28%), overall cytotoxicity was lower in *Y*. *pestis*-infected cells (S2D Fig; 12% vs. 4%). Similarly, *Y*. *pestis* did not induce elevated permeability or cytotoxicity in human neutrophils (S2E and S2F Fig). These data demonstrate that while neutrophils can rapidly synthesize LTB_4_ in response to other bacterial pathogens, neither murine nor human neutrophils appear to synthesize LTB_4_ in response to *Y*. *pestis*.

### *Y*. *pestis* actively inhibits LTB_4_ synthesis

**Fig 5:**
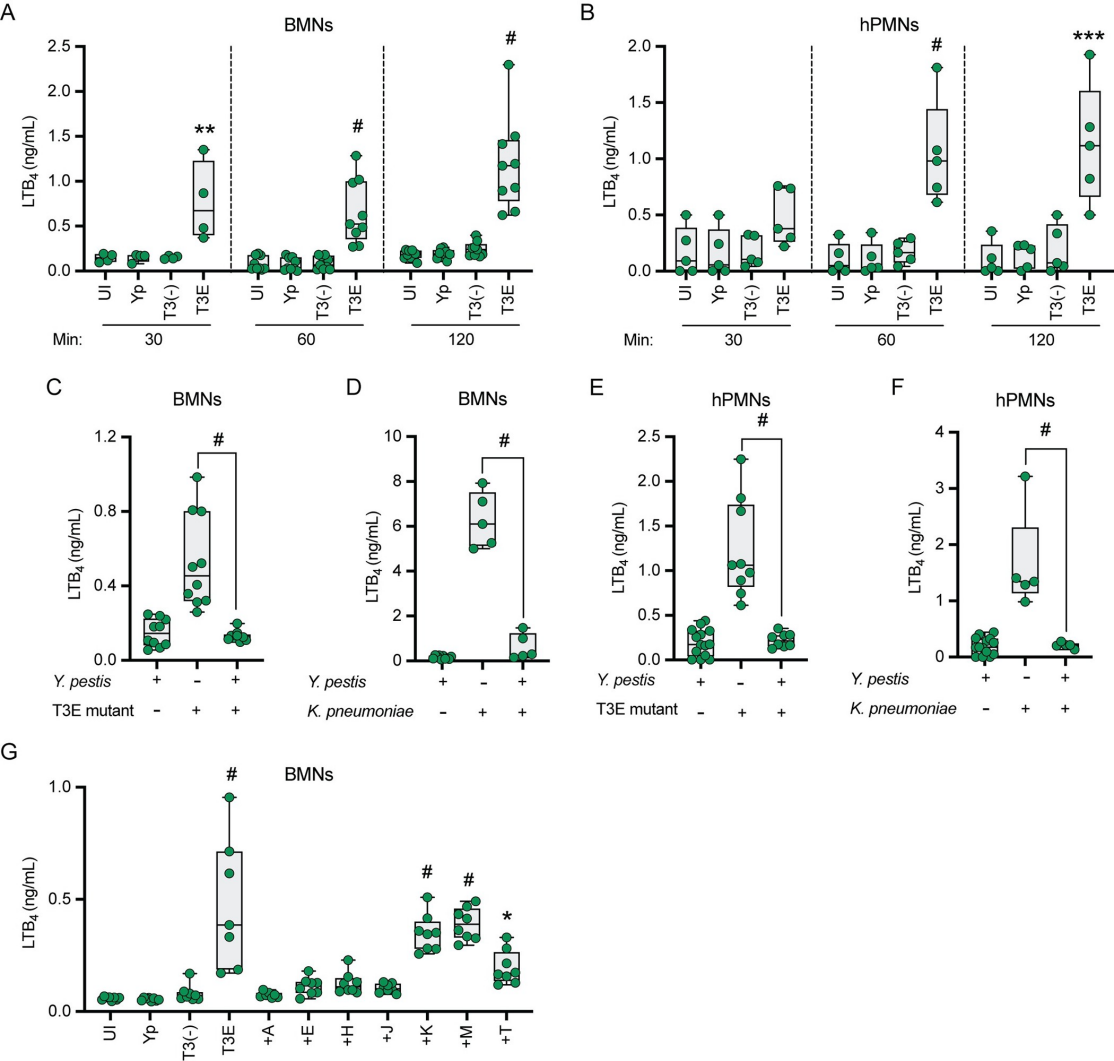
*Y*. *pestis* actively inhibits LTB_4_ synthesis. (A) Murine (BMNs) or (B) human (hPMNs) neutrophils were infected with *Y*. *pestis* (Yp) or mutants that either lacked the Yop effectors (T3E) or the Yop effectors and the T3SS [T3(-)] at an MOI of 20 and LTB_4_ was measured at 30, 60, or 120 min. (C and D) Murine (BMNs) or (E and F) human (hPMNs) neutrophils were co-infected with the indicated bacteria at a combined MOI of 20 and LTB_4_ was measured at 60 min post-infection. (G) Murine neutrophils (BMNs) were infected with Yp, T3E, T3(-), or *Y*. *pestis* strains expressing only one Yop effector (+A = YpkA; +E = YopE; +H = YopH; +J = YopJ; +K = YopK; +M = YopM; or +T = YopT) at an MOI of 20 and LTB_4_ was measured at 60 min post-infection. Each symbol represents an independent biological infection and the box plot represents the median of the group ± the range. UI = uninfected. One-way ANOVA with Dunnett’s *post hoc* test compared to uninfected for A, B, and G, or Tukey’s *post hoc* test compared to each condition for C, D, E, and F. * = p ≤ 0.05, ** = p ≤ 0.01, *** = p ≤ 0.001, # = p ≤ 0.0001.

Seven Yop effectors are secreted via the T3SS [21–24], and Pulsifer et al. previously showed Yop effector-mediated inhibition of LTB_4_ synthesis in human neutrophils by YpkA, YopE, YopJ, YopH, and YopT at an MOI of 100 [26]. However, Yop inhibition of LTB_4_ synthesis by murine neutrophils has not been previously investigated, nor whether the same Yop effectors are sufficient to inhibit LTB_4_ synthesis at a lower MOI. Therefore, murine and human neutrophils were infected at an MOI of 20 with a *Y*. *pestis* mutant strain that expresses the T3SS but lacks all seven Yop effectors (*Y*. *pestis* T3E) [50]. In contrast to *Y*. *pestis* infected cells, we observed a significant increase in LTB_4_ synthesis in response to the *Y*. *pestis* T3E strain, indicating that the Yop effectors inhibit synthesis (Fig 5A and 5B; p ≤ 0.0001). Moreover, when neutrophils were simultaneously infected with *Y*. *pestis* and the *Y*. *pestis* T3E mutant or *Y*. *pestis* and the *K*. *pneumoniae manC* mutant, LTB_4_ levels were significantly lower than *Y*. *pestis* T3E or *K*. *pneumoniae* *manC* only infections (Fig 5C–5F; p ≤ 0.0001). To determine if individual Yop effectors were sufficient to inhibit synthesis, murine neutrophils were infected with *Y*. *pestis* strains that expressed only one Yop effector [50]. LTB_4_ synthesis was significantly decreased if *Y*. *pestis* expressed YpkA, YopE, YopH, or YopJ, and an intermediate phenotype was observed during infection with a strain expressing YopT (Fig 5G). These phenotypes recapitulated those previously reported for human neutrophils [26]. Together these data confirm that *Y*. *pestis* is not simply evading immune recognition but is actively inhibiting LTB_4_ synthesis via the activity of multiple Yop effectors.

### Neutrophils synthesize LTB_4_ in response to the *Y*. *pestis* T3SS in the absence of the Yop effectors

**Fig 6:**
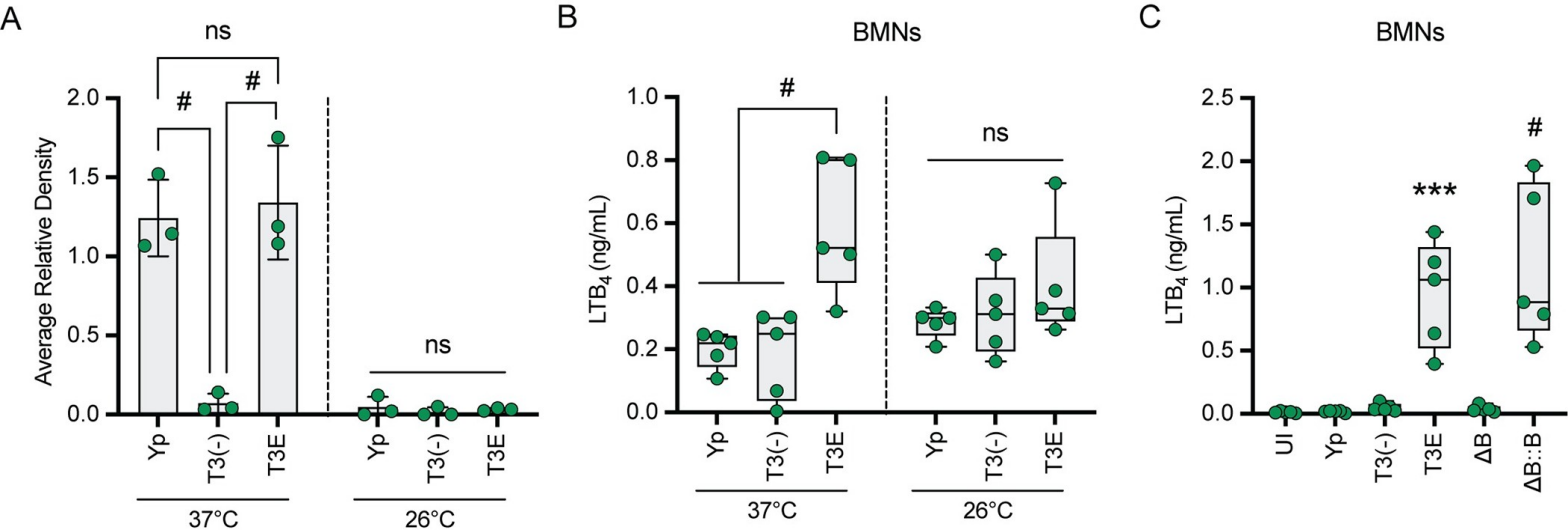
Neutrophils synthesize LTB_4_ in response to the *Y*. *pestis* T3SS in the absence of the Yop effectors. (A) Relative expression of LcrV based on western blots normalized to total protein loaded from bacteria cultured at 37 or 26°C. (B) Murine neutrophils (BMNs) were infected (MOI of 20) with *Y*. *pestis* (Yp) or mutants that either lacked the Yop effectors (T3E) or the Yop effectors and the T3SS [T3(-)] cultured at 37 or 26°C and LTB_4_ was measured at 60 min post-infection. (C) Murine neutrophils (BMNs) were infected (MOI of 20) with Yp, T3E, T3(-), a *yopB* mutant in the T3E background (ΔB), or ΔB complemented with *yopB* (ΔB::B) cultured at 37°C and LTB_4_ was measured at 60 min post-infection. Each symbol represents an independent biological infection and the box plot represents the median of the group ± the range. One-way ANOVA with Tukey’s *post hoc* test compared to each condition for A and B and Dunnett’s *post hoc* test compared to uninfected for C. ns = not significant, *** = p ≤ 0.001, # = p ≤ 0.0001.

Components of the T3SS are pathogen-associated molecular patterns (PAMPs) that are recognized by innate immune cells [7,51,52], suggesting that T3SS interactions with neutrophils may be responsible for the synthesis of LTB_4_ during infections with the *Y*. *pestis* T3E strain. To test this hypothesis, we infected murine and human neutrophils with a *Y*. *pestis* strain lacking the pCD1 plasmid encoding the entire Ysc T3SS [*Y*. *pestis* T3^(-)^]. Unlike infections with *Y*. *pestis* T3E, we did not observe an increase in LTB_4_ synthesis by neutrophils during interactions with *Y*. *pestis* T3^(-)^ compared to uninfected or *Y*. *pestis* infected cells, even after 2 h of infection (Fig 5A and 5B). Importantly, *Y*. *pestis* T3^(-)^ infection did not appear to result in increased neutrophil cell permeability or cytotoxicity (S2 Fig). To independently test that the T3SS is required to induce LTB_4_ synthesis, *Y*. *pestis* T3E was cultured under conditions that alter the expression of the T3SS prior to infection of neutrophils [5,8,16]. Measuring expression of the LcrV protein as a proxy for overall T3SS expression confirmed decreased T3SS expression in cultures grown at 26°C compared to 37°C (Figs 6A and S3A). As predicted by the *Y*. *pestis* T3^(-)^ data, LTB_4_ synthesis was not observed from neutrophils infected with *Y*. *pestis* T3E strains grown at 26°C, while synthesis was induced from bacteria cultured at 37°C (Fig 6B). No difference in bacterial viability between any of the *Y*. *pestis* strains was observed during the time frame of the experiment, diminishing the possibility that differences in neutrophil killing of the bacteria was responsible for these phenotypes (S3B Fig). Finally, neutrophils were infected with a *Y*. *pestis* T3E *yopB* mutant, which retains the other pCD1 encoded genes, but is defective in expression of the translocase that directly interacts with the host cell and is required for injection of the effector proteins [8,53–56]. Similar to the *Y*. *pestis* T3^(-)^ strain, the *Y*. *pestis* T3E *yopB* mutant did not induce LTB_4_ synthesis, but synthesis was restored by *yopB* complementation (*yopB*::c*yopB*) (Fig 6C). Together, these data indicate that neutrophils recognize components of the *Y*. *pestis* T3SS as PAMPs, leading to the induction of LTB_4_ synthesis, but only in the absence of the Yop effectors.

### *Y*. *pestis* inhibition of LTB_4_ synthesis is conserved during interactions with other leukocytes

**Fig 7:**
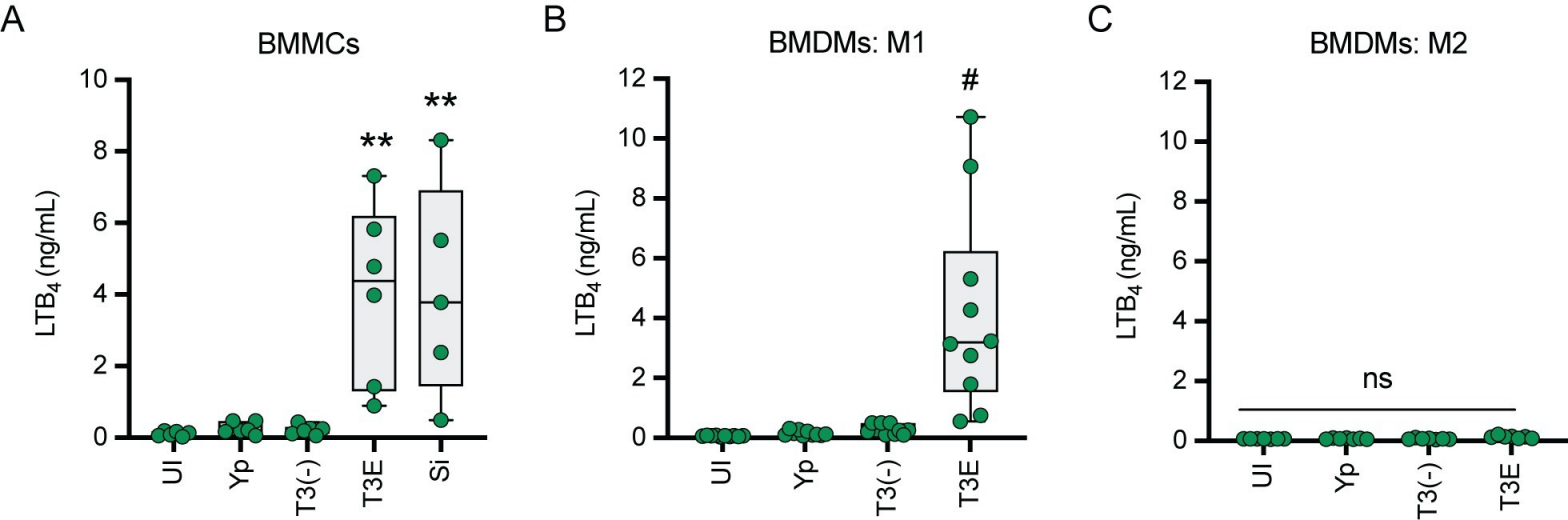
Lack of LTB_4_ response to *Y*. *pestis* is conserved in other leukocytes. (A) Murine bone-marrow derived mast cells (BMMCs) were infected with *Y*. *pestis* (Yp) or with mutants that either lacked the Yop effectors (T3E) or the Yop effectors and the T3SS [T3(-)] at an MOI of 20, or treated with 100 mg/cm^2^ crystalline silica (Si) and LTB_4_ was measured at 2 h post-infection. (B and C) Murine bone-marrow derived macrophages (BMDMs) differentiated towards (B) M1 or (C) M2 phenotypes were infected with Yp, T3E, or T3(-) at an MOI of 20 and LTB_4_ was measured at 4 h post-infection. UI = uninfected. Each symbol represents an independent biological infection and the box plot represents the median of the group ± the range. One-way ANOVA with Dunnett’s post hoc test compared to uninfected. ** = p ≤ 0.01, # = p ≤ 0.0001, ns = not significant.

In addition to neutrophils, two other lung resident leukocytes that can produce LTB_4_ are mast cells and macrophages [33]. To determine if *Y*. *pestis* inhibits LTB_4_ synthesis by these two cell types, bone marrow-derived mast cells and macrophages were isolated from C57BL/6J mice and infected with *Y*. *pestis*, *Y*. *pestis* T3E, or *Y*. *pestis* T3^(-)^. We observed no synthesis of LTB_4_ by mast cells, even after 2 h of interacting with *Y*. *pestis* (Fig 7A). However, LTB_4_ synthesis was significantly elevated in the absence of the Yop effectors (Fig 7A; p ≤ 0.01), reaching levels similar to that of mast cells stimulated with crystalline silica, a potent inducer of LTB_4_ synthesis [57,58]. LTB_4_ synthesis by mast cells was also dependent on the presence of the T3SS, as the *Y*. *pestis* T3^(-)^ strain did not induce LTB_4_ synthesis (Fig 7A). For macrophages, previous reports indicate that polarization influences the ability to produce LTB_4_, with M1-polarized macrophages better able to synthesize LTB_4_ in response to bacterial ligands than M2-polarized cells [59]. Therefore, we measured LTB_4_ synthesis of both M1- and M2-polarized macrophages (S4 Fig). Again, we observed no significant synthesis of LTB_4_ by either macrophage population during interactions with *Y*. *pestis*, even after 4 h post-infection (Fig 7B and 7C). However, significant synthesis of LTB_4_ was observed in M1-polarized macrophages in response to the *Y*. *pestis* T3E strain, which was dependent on the presence of the T3SS (Fig 7B; p ≤ 0.0001). As suggested by previous reports [60], we did not observe significant changes in LTB_4_ synthesis by M2-polarized macrophages during interactions with any of the *Y*. *pestis* strains tested (Fig 7C). Together, these data indicate that mast cells and M1-polarized macrophages can synthesize LTB_4_ in response to the *Y*. *pestis* T3SS, but the activity of the Yop effectors inhibits this response.

## Discussion

**Fig 8:**
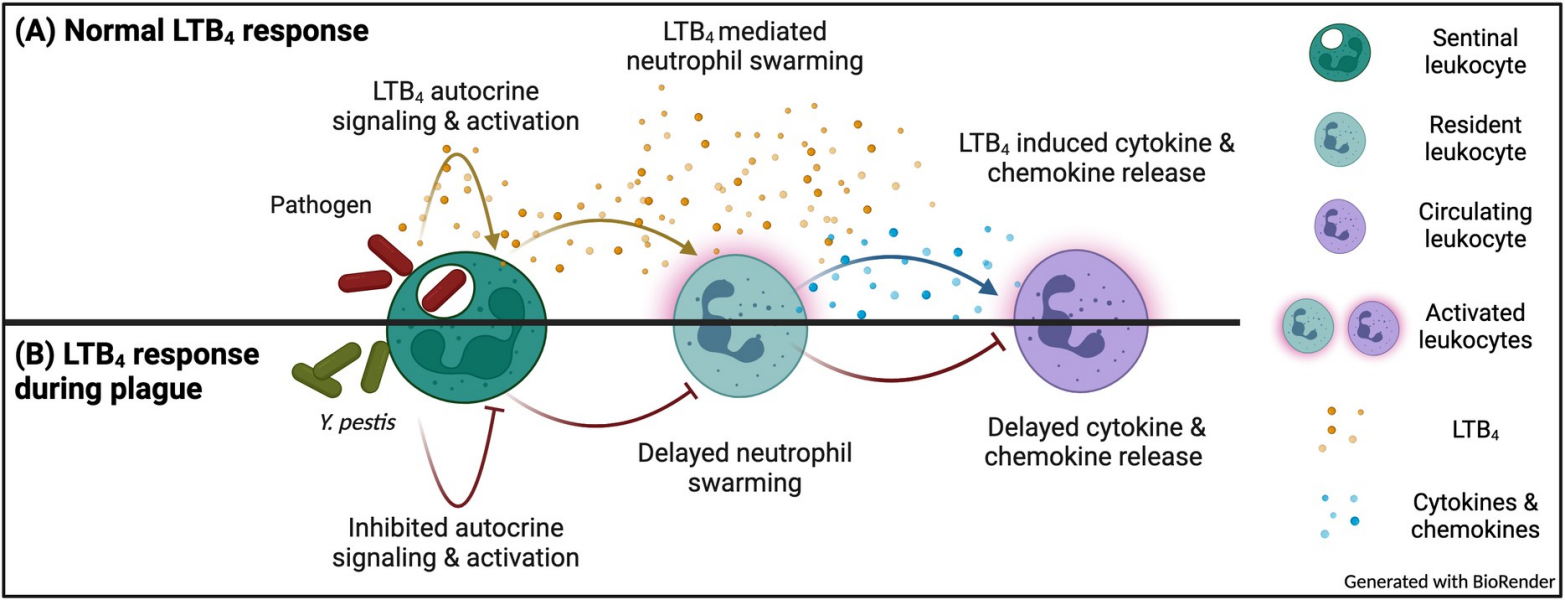
Working model for inhibition of the inflammatory cascade during plague. (A) Normal response by sentinel leukocytes results in rapid production of LTB_4_ that leads to autocrine signaling, neutrophil swarming, and induction of cytokine and chemokine release. (B) *Y*. *pestis* inhibits the production of LTB_4_ via the action of the Yop effectors, which delays resident neutrophil recruitment and subsequent production of cytokines and chemokines needed for inflammation.

A hallmark manifestation of plague is the absence of inflammation during the early stages of infection, which is critical to *Y*. *pestis* virulence [10,17,28,52]. While *Y*. *pestis* has been shown to actively dampen the host immune response, there is a gap in our understanding of the role of lipid mediators of inflammation during plague. This study sought to better define the host inflammatory lipid mediator response during pneumonic plague and expands our current understanding of how *Y*. *pestis* manipulates the immune system. During the earliest stages of infection, the host appears unable to initiate a timely LTB_4_ response (Fig 1). Moreover, we demonstrated that exogenous treatment with LTB_4_ can alter the host response to *Y*. *pestis* (Fig 3), suggesting that LTB_4_ manipulation by *Y*. *pestis* contributes to disease outcome. Because LTB_4_ is a potent chemoattractant crucial for rapid inflammation [31,32,61], a delay in LTB_4_ synthesis during plague likely has a significant impact on the ability of the host to mount a robust inflammatory response needed to inhibit *Y*. *pestis* colonization. First, in the absence of LTB_4_, sentinel leukocytes will not undergo autocrine signaling via LTB_4_-BLT1. Because LTB_4_-BLT1 engagement activates antimicrobial programs in leukocytes [31,32,46,62–64], the absence of autocrine signaling diminishes the ability of sentinel leukocytes directly interacting with *Y*. *pestis* to mount an effective antimicrobial response to kill the bacteria. LTB_4_ synthesis is also regulated by BLT1 signaling, and autocrine signaling is required to amplify the production of LTB_4_ needed to rapidly recruit additional tissue-resident immune cells to the site of infection [31,32,63,65,66]. Therefore, the normal feed-forward amplification of LTB_4_ synthesis, which is key for a rapid response to a bacterial infection, will also be inhibited by *Y*. *pestis*. Second, because LTB_4_ is required for neutrophil swarming [65,67,68], *Y*. *pestis* will also inhibit this key inflammatory mechanism [69]. Neutrophil swarming is required to contain bacteria at initial sites of infection [70,71]. Thus, while individual neutrophils may migrate towards sites of *Y*. *pestis* infection, effective neutrophil swarming of large populations of neutrophils will be diminished. Finally, LTB_4_ is a diffusible molecule that can induce the inflammatory cascade in bystander cells [32,72]. Thus, while *Y*. *pestis* can inhibit cytokine and chemokine expression by cells with which it directly interacts [11,15], inhibition of LTB_4_ synthesis likely also delays subsequent release of molecules by cells that do not directly interact with the bacteria. Together with the bacteria’s other immune evasion mechanisms, inhibition of LTB_4_ synthesis is likely another significant contributor to the generation of the non-inflammatory environment associated with the early stages of pneumonic plague [10,11,15]. Incorporating these new LTB_4_ data with published findings from other laboratories [10,11,15], we have updated our working model of *Y*. *pestis* inhibition of inflammation during pneumonic plague (Fig 8).

These studies also revealed that components of the T3SS trigger LTB_4_ synthesis by leukocytes. Because our previous work with human samples indicated that neutrophils synthesize LTB_4_ in response to *Y*. *pestis* in the absence of the T3SS [26], we were initially surprised that we did not observe LTB_4_ synthesis by murine neutrophils to the *Y*. *pestis* T3^(-)^ strain. However, when we infected human neutrophils with lower MOIs, we observed that they also did not synthesize LTB_4_ in the absence of the T3SS (Fig 5B). Under these infection conditions, neutrophils from both species only produced LTB_4_ in response to *Y*. *pestis* expressing the T3SS but none of the Yop effectors. These data support that components of the T3SS are PAMPs produced by *Y*. *pestis* that are not only recognized by macrophages [73] but also by neutrophils. While previous studies have indicated that the Yop effectors are PAMPs in neutrophils [74,75], to our knowledge the data presented here represent the first example that non-effector components of the *Y*. *pestis* T3SS can also be recognized as a PAMP by neutrophils. In macrophages, in the absence of the Yop effectors, interactions with the T3SS, notably the translocon proteins YopB and YopD, induce NLRP3-dependent activation of the caspase 1 inflammasome, IL1-β secretion, and pyroptosis [54,76], suggesting that inflammasome activation may contribute to LTB_4_ synthesis during interactions with *Y*. *pestis* T3E. However, whether inflammasome activation is required for the *Y*. *pestis* T3SS-mediated LTB_4_ synthesis remains unclear, as LTB_4_ synthesis in response to other stimuli is not dependent on inflammasome activation [57,77,78]. Interestingly, infection of neutrophils with a strain of *Y*. *pestis* that only expresses YopK, which has been reported to inhibit NLRP3 inflammasome activation in macrophages [53,54], does not inhibit LTB_4_ synthesis (Fig 4C) [26], supporting the possibility that LTB_4_ synthesis may not be dependent on inflammasome activation in neutrophils. Future studies using neutrophils from mice defective in specific NLRs and caspases will allow us to definitively determine if inflammasome activation is required for LTB_4_ synthesis in response to the *Y*. *pestis* T3SS. We have also confirmed that four Yop effectors, YpkA, YopE, YopJ, and YopH are sufficient to inhibit LTB_4_ synthesis by both human and murine neutrophils. Synthesis of LTB_4_ requires MAPK- and Ca^2+^-dependent activation of cPLA2 and 5-LOX [34,79]. Previous work, primarily in macrophages, has shown that both of these signaling pathways are efficiently inhibited by these four Yop effectors [8,20,80–84], suggesting that subversion of MAPK and Ca^2+^ signaling by *Y*. *pestis* is responsible for inhibition of LTB_4_ synthesis. Supporting this hypothesis, Pulsifer et al. demonstrated that inhibition of ERK phosphorylation by YopJ is sufficient to inhibit LTB_4_ synthesis by human neutrophils [26]. Defining the specific molecular mechanisms employed by YpkA, YopE, and YopH to inhibit LTB_4_ synthesis will be important in better understanding *Y*. *pestis* virulence and is a long-term goal.

One of the key antimicrobial mechanisms inhibited by the Yop effectors is phagocytosis [5,16,85,86], and Hedge et al. have previously shown that phagocytosis of crystalline silica is required for LTB_4_ synthesis in that model of sterile inflammation [57]. These data raise the possibility that inhibition of phagocytosis by *Y*. *pestis* may not only inhibit bacterial killing, but LTB_4_ synthesis and rapid initiation of inflammatory programing in neutrophils. Studies to delineate the contribution of phagocytosis to LTB_4_ synthesis are ongoing, but the differences in LTB_4_ synthesis by cells infected with *Y*. *pestis* T3E and *Y*. *pestis* T3^(-)^ suggest that phagocytosis alone is not sufficient to trigger LTB_4_ synthesis in the absence of proper PAMPs, in this case components of the T3SS. Moreover, the lack of LTB_4_ synthesis in response to *Y*. *pestis* T3^(-)^ also differed from what we observed for other gram-negative bacteria without a T3SS (*E*. *coli* and *K*. *pneumoniae*), indicating that *Y*. *pestis* may also mask other potential gram-negative PAMPS that would typically be recognized by neutrophils. These data support that *Y*. *pestis* has evolved both active (via the Yop effectors) and passive mechanisms to evade immune recognition and induction of LTB_4_ synthesis. It is worth noting that unlike human neutrophils, murine neutrophils did not appear to synthesize LTB_4_ during infections with the T3^(-)^ strain at high MOIs (S5 Fig). Differences in neutrophil responses between the two species have been well documented [87–91], but these observations merit further investigation into LTB_4_ responses by human neutrophils using higher MOIs to determine if human neutrophils are able to recognize other PAMPs during *Y*. *pestis* infection.

Finally, while we focused primarily on LTB_4_ in this study, we also observed changes in the synthesis of other lipids during plague that merit future considerations (S1 Table). The rapid cyclooxygenase response raises questions about whether prostaglandins are protective or detrimental during pneumonic plague. Historically, prostaglandins were thought to promote inflammation, but these mediators appear more nuanced under closer scrutiny and can just as likely inhibit inflammation, as well as participate in normal development physiology, without eliciting inflammation [42,43,92]. The prostaglandins we observed as being significantly elevated during the non-inflammatory stage of pneumonic plague—PGA_2_, PGD_2_, PGE_2_, and PGJ_2_—have been shown to inhibit inflammation in various models, especially as concentrations increase [42,43,93–95]. PGE_2_ can inhibit NADPH oxidase activity during infection with *K*. *pneumoniae*, which suppressed bacterial killing [96], and directly counteracts the proinflammatory activities of LTB_4_ [97,98]. The phagocytic index of LTB_4_-stimulated rat alveolar macrophages (AMs) is reduced when co-stimulated with PGE_2_ [98]. Moreover, AMs treated with PGE_2_ showed a 40% reduction in LTB_4_ synthesis when stimulated with an ionophore known to induce a strong LTB_4_ response [97]. This inhibition of LTB_4_ by PGE_2_ is suspected to be via an increase in second messenger cAMP that activates protein kinase A (PKA), which has been shown to inhibit LTB_4_ synthesis [97,99]. Together, these data suggest that the elevated levels of prostaglandin synthesis observed during pneumonic plague may contribute to the blunted LTB_4_ response by the host.

In conclusion, we have defined the kinetics of the key inflammatory lipid mediator LTB_4_ during pneumonic plague, which revealed a blunted response during the early stages of infection. Furthermore, we have shown that *Y*. *pestis* actively manipulates LTB_4_ synthesis by leukocytes via the activity of Yop effectors to generate a beneficial inflammatory outcome to the pathogen. These discoveries warrant further research into the role of lipids, and subsequent manipulation of their synthesis by *Y*. *pestis*, to fully understand the molecular mechanisms *Y*. *pestis* has evolved to manipulate the mammalian immune response.

## Material and methods

### Ethics statement

All animal work was approved by the University of Louisville Institutional Animal Care and Use Committee (IACUC Protocol #22157). Use of human neutrophils was approved by the University of Louisville Institutional Review Board guidelines (IRB #96.0191) and written consents for use were obtained.

### Bacterial strains

Bacterial strains used in this study are listed in S2 Table. For mouse infections, *Y*. *pestis* was grown at 26°C for 6–8 h, diluted to an optical density (OD) (600 nm) of 0.05 in Bacto brain heart infusion (BHI) broth (BD Biosciences Cat. No. 237500) with 2.5 mM CaCl_2_ and then grown at 37°C with aeration for 15–18 h [100]. For cell culture infections, *Y*. *pestis* was cultured with BHI broth for 15–18 h at 26°C in aeration. Cultures were then diluted 1:10 in fresh, warmed BHI broth containing 20 mM MgCl_2_ and 20 mM Na-oxalate and cultured at 37°C for 3 h with aeration to induce expression of the T3SS. Bacterial concentrations were determined using a spectrophotometer and diluted to desired concentrations in 1 × Dulbecco’s phosphate-buffered saline (DPBS) for mouse infections or fresh medium for *in vitro* studies. Concentrations of bacterial inoculums for mouse studies were confirmed by serial dilution and enumeration on BHI agar plates.

### Mouse infections

All animal work was performed at least twice to ensure reproducibility. 6–8 week-old C57BL/6J or BLT1^-/-^ [49] male and female mice were infected with *Y*. *pestis* KIM5+ or *Y*. *pestis* CO92 LUX_p*cysZK*_. For lipid measurements, mice were anesthetized with ketamine/xylazine and administered 20 μL of *Y*. *pestis* KIM5+ suspended in 1× DPBS to the left nare as previously described [48,100]. Mice were monitored for the development of moribund disease symptoms twice daily and humanely euthanized when they met previously approved end point criteria. At 6, 12, 24, 36, or 48 h, mice were humanely euthanized by CO_2_ asphyxiation and lungs were harvested and lung masses recorded. Lungs were transferred to a 2 mL tube pre-filled with 2.8 mm ceramic beads (VWR, Cat. No. 10158–612), flash frozen on dry ice, and stored at -80°C until preparation for lipid analysis. For CFU studies, mice were humanely euthanized by CO_2_ asphyxiation at 12 or 24 h and lungs were harvested. Lungs were transferred to Whirl Pak’s containing 1 mL of 1 x DPBS, and gently homogenized using a serological pipette. Homogenized tissues were serial diluted and plated onto BHI agar. After 2 days of incubation at 26°C, bacteria were enumerated. For optical imaging and survival curves, mice were infected with *Y*. *pestis* CO92 LUX_p*cysZK*_ and monitored for bacterial proliferation as a function of bioluminescence by optical imaging and for the development of moribund disease. At each time point, mice were anesthetized with isoflurane and imaged using the IVIS Spectrum imaging system (Caliper Life Sciences, Hopkinton, MA). Average radiance (photons/s/cm2) was calculated for the lungs as previously described [48]. For the exogenous LTB_4_ treatment, mice were intraperitoneally injected with 1 x DPBS or 10 nmol LTB_4_ (Cayman Chemical Cat. No. 20110). At 1 h post-treatment, mice were administered 10^5^ CFU of *Y*. *pestis* KIM5+ via intraperitoneal injection. At 3 h post infection, mice were humanely euthanized, and the peritoneal cavity was washed and collected using 2 lavages of 1 mL of 1 x DPBS. Lavages were used for CFU enumeration or neutrophil quantification by flow cytometry.

### Lipid extraction and quantification by LC-MS

To quantify LTB_4_ abundance from whole lungs, lungs were thawed with 1.8 mL of ice cold 75% methanol + 0.1% BHT for 3 minutes. Lungs were then homogenized with a Bead Ruptor 4 (OMNI) at speed 5 (5 m/s) for 4 cycles of 45 seconds with 1-minute pauses in which the lungs were placed on ice. Tissue debris was then centrifuged for 10 min at 1,500 x g at 4°C. The supernatant (~1.5 mL) was then transferred to a fresh eppendorf tube, incubated at 4°C for 24 h to inactivate *Y*. *pestis* and extract lipids. After successful inactivation, samples were removed from BSL3 containment and stored at -80°C. Lipid extraction was then performed as previously described [101]. For the expanded global lipid analysis, lungs were thawed with 1.5 mL of ice cold 1 x DPBS + HALT protease and phosphatase inhibitor cocktail for 3 minutes. Lungs were then homogenized with a Bead Ruptor 4. Tissue debris was then centrifuged for 10 min at 1,500 x g at 4°C. The supernatant (~1.5 mL) was then transferred to a fresh eppendorf tube. From this, 250 μL of supernatant was combined with 750 μL of 100% methanol + 0.1% BHT (final concentration of 75%) and incubated at 4°C for 24 h to inactivate *Y*. *pestis* and extract lipids. After confirmation of successful inactivation of *Y*. *pestis*, lipids were extracted and quantified by the Wayne State University Lipidomics Facility as previously described [102]. The extracted samples were analyzed for the fatty acyl lipidome using standardized methods as described previously [103,104].

### Flow cytometry

To quantify the neutrophil population from peritoneal lavages, cells were labeled with anti-Ly6G antibody (1:400; BD Pharminogen Cat. No. 551460) and anti-CD11b antibody (1:600; Biolegend Cat. No. 101212) for 1 h on ice, in the dark. Cells were pelleted and resuspended in 1% PFA. Single cell suspensions were generated by straining with 70 μM mesh prior to analysis on the flow cytometer. Neutrophils were identified as cells with high expression of Ly6G and CD11b and data is represented as the percent of the population that were classified as neutrophils. An example of the gating strategy is shown in S1 Fig.

### Cell isolation and cultivation

Human neutrophils were isolated from the peripheral blood of healthy, medication-free donors, as described previously [105]. Briefly, white blood cells were isolated from whole blood using a 6% dextran solution. Neutrophils were then separated from monocytes using a percoll gradient of 42% and 50.5%. RBCs were then lysed from the neutrophil containing layer using 0.2% NaCl for 30 seconds and followed by a quench with 5 mL 1.6% NaCl. Neutrophil isolations yielded ≥ 95% purity and were used within 1 h of isolation. Murine neutrophils were isolated from bone marrow of 7-12-week-old mice using an Anti-Ly-6G Microbeads kit (Miltenyi Biotec Cat. No. 130-120-337) per the manufacturer’s instructions. Neutrophil isolations yielded ≥ 95% purity and were used within 1 h of isolation. Macrophages were differentiated from murine bone marrow in DMEM supplemented with 1 mM Na-pyruvate and 10% FBS for 6 days. Macrophages were either polarized with 10 ng/mL of GM-CSF (M1; Kingfisher Biotech Cat. No. RP0407M) or with 30% L929 conditioned media and 10 ng/mL of M-CSF (M2; Kingfisher Biotech Cat. No. RP0462M) throughout the differentiation. The medium was replaced on days 1 and 3 (adapted from [106]). Polarization was confirmed by qRT-PCR, as previously described [107], using markers for M1 and M2 phenotypes, TNF-α and Il-10, respectively (S4 Fig). Murine mast cells were isolated and differentiated from bone marrow as previously described [108]. Briefly, isolated bone marrow cells were resuspended in BMMC culture medium [DMEM containing 10% FCS, penicillin (100 units/mL), streptomycin (100 mg/mL), 2 mmol/L L-glutamine, and 50 mmol/L β-mercaptoethanol] supplemented with recombinant mouse stem cell factor (SCF) (12.5 ng/mL; R&D Systems Cat. No. 455-MC) and recombinant mouse IL-3 (10 ng/mL; R&D Systems Cat. No. 403-ML). Cells were plated at a density of 1 x 10^6^ cells/mL in a T-75 cm^2^ flask. Nonadherent cells were transferred after 48 hours into fresh flasks without disturbing the adherent (fibroblast) cells. Mast cells were visible after 4 weeks of culture and propagated further or plated for experiments in DMEM without antibiotics.

### Leukocyte infections

Human neutrophils were resuspended in Kreb’s buffer (w/ Ca^2+^ and Mg^2+^) then adhered to 24-well plates for 30 min that were coated with pooled human serum prior to infection (wells were washed twice with 1 x DPBS prior to plating the cells). Murine bone marrow neutrophils were resuspended in RPMI + 5% FBS then adhered to 24-well plates for 30 min that were coated with FBS prior to infection (wells were washed twice with 1 x DPBS prior to plating the cells). Neutrophils were infected at a multiplicity of infection (MOI) of 20, 50, or 100 and incubated for 1 h in a cell culture incubator at 37°C with a constant rate of 5% CO_2_. Co-infections were performed at a final MOI of 20 (MOI of 10 for each strain). 1 h post-infection, supernatants were collected, centrifuged for 1 min at 6,000 x g, and supernatants devoid of cells were transferred to a fresh eppendorf tube. Macrophages were adhered to 24-well plates in DMEM + 10% FBS 1 day prior to infection. Macrophages were infected at an MOI of 20. At 4 h post-infection, supernatants were collected, centrifuged for 1 min at 6,000 x g, and supernatants devoid of cells were transferred to a fresh eppendorf tube. Mast cells were adhered to 24-well plates in DMEM only for 1 h prior to infection. Mast cells were infected at an MOI of 20 or treated with crystalline silica (100 mg/cm^2^). At 2 h post-infection supernatants were collected, centrifuged for 1 min at 6,000 x g, and supernatants devoid of cells were transferred to a fresh eppendorf tube. All infections were synchronized by centrifugation (200 x g for 5 min). All samples were stored at -80°C until ELISA.

### Measurement of LTB_4_ by enzyme-linked immunosorbent assay

Supernatants of neutrophils, macrophages, and mast cells were collected and measured for LTB_4_ by ELISA per manufacturer’s instructions (Cayman Chemicals Cat. No. 520111).

### Cell viability assays

To determine leukocyte permeability, cells were incubated with trypan blue for 5 min and trypan blue exclusion was measured using SD100 counting chambers (VWR Cat. No. MSPP-CHT4SD100) and a cell counter (Nexcelom Cellometer Auto T4). To determine leukocyte cytotoxicity, lactate dehydrogenase (LDH) was measured from leukocyte supernatants using the CytoTox 96 Non-Radioactive Cytotoxicity kit (Promega Cat. No. g1780) per the manufacturer’s instructions.

### Bacterial viability assays

To measure bacterial viability during interactions with neutrophils, murine neutrophils were resuspended in RPMI + 5% FBS then adhered to 96-well white bottom plates for 30 min coated with FBS prior to infection (wells were washed twice with 1 x DPBS prior to plating the cells). Neutrophils were infected at an MOI of 20, centrifuged for 5 min at 200 x g, and bacterial viability was measured as a function of bioluminescence using a plate reader (BioTek Cytation 1 imaging reader).

### Measurement of LcrV by western blot

Bacterial strains were cultured with BHI broth for 15–18 h at 26°C in aeration. Cultures were then diluted 1:10 in fresh warmed BHI broth containing 20 mM MgCl_2_ and 20 mM Na-oxalate and cultured at 37 or 26°C for 3 h. 1 OD_600_ of bacterial pellets were collected and resuspended in 1 x SDS-PAGE loading buffer, boiled for 10 min, and 0.1 OD_600_ was separated on a 10% SDS-PAGE gel. As a positive control, 0.2 g of recombinant LcrV protein was used (BEI resources Cat. No. NR-32875). Samples were immunoblotted with polyclonal anti-LcrV antibody diluted to 1:4,000 (BEI Resources Cat. No. NR-31022). Anti-goat IgG HRP secondary antibody was diluted to 1:5,000 (Bio-Techne Cat. No. HAF017). Densitometry was performed using ImageJ software to compare LcrV bands between samples [109].

### Statistics

For all studies, male and female mice or human donors were used and no sex biases were observed for any phenotype. All *in vivo* experiments were repeated at least twice and *in vitro* experiments at least 5 times. Where noted in the figure legends, figures may represent the combined data from multiple biologically independent experiments. For *in vitro* experiments, each data point represents data from biologically independent experiments performed on different days. Where appropriate and as indicated in the figure legends, statistical comparisons were performed with Prism (GraphPad) using one-way analysis of variance (ANOVA) with Dunnett’s or Tukey’s *post hoc* test, T-test with Mann-Whitney’s *post hoc* test, or Log-Rank analysis. P values ≤ 0.05 were considered statistically significant and reported. For LC-MS analysis of lipids, a LIMMA—Moderated T-test was performed using a modified version of a previously published protocol using R packages [110–112]. Briefly, raw data were transformed by taking logarithmic base 2 followed by quantile normalization. Missing values were then ascribed using a singular value decomposition method. Lipids missing > 40% of the values were excluded from subsequent analysis. Finally, differentially abundant lipids (p ≤ 0.05) were further filtered by fold-change (FC) criteria (1 < log_2_FC < 1) and multiple comparisons testing with a false discovery rate.

## Supporting information

S1 Fig Gating strategy for identifying neutrophil populations in the peritoneal cavity. Example gating strategy from the PBS-treated group from Fig 3A.
(TIF)

S2 Fig Absence of LTB_4_ response to *Y*. *pestis* is not due to cell death. (A-D) Murine (BMNs) or (E-F) human (hPMNs) neutrophils were infected with *Y*. *pestis* (Yp) or mutants that either lacked the Yop effectors (T3E) or lacked the Yop effectors and the T3SS [T3(-)] at the indicated MOIs and cell permeability as a function of trypan exclusion or cytotoxicity as a function of LDH release was measured at 1 h post-infection. Each symbol represents an independent biological infection and the box plot represents the median of the group ± the range. UI = uninfected. One-way ANOVA with Dunnett’s *post hoc* test compared to uninfected. * = p ≤ 0.05, ** = p ≤ 0.01, *** = p ≤ 0.001, # = p ≤ 0.0001.
(TIF)

S3 Fig Expression of T3SS needle required for LTB_4_ synthesis in response to *Y*. *pestis*. (A) Representative western blot and Coomassie images of *Y*. *pestis* lysates (0.1 OD; 1 OD = 3 x 10^8^ CFU) harvested from cultures grown at 37°C or 26°C used for densitometry reported in Fig 6A. (B) Bacterial viability measured by a function of bioluminescence after 1 h infection of neutrophils. + or Yp = *Y*. *pestis*,— or T3(-) = *Y*. *pestis* T3(-); E or T3E = *Y*. *pestis* T3E; LcrV = 0.2 μg recombinant LcrV protein. Each symbol represents an independent biological infection and the bar graph represents the mean ± the standard deviation. One-way ANOVA with Tukey’s *post hoc* test compared to each condition. ns = not significant.
(TIF)

S4 Fig M1 polarization required for macrophage synthesis towards *Y*. *pestis* T3SS. qRT-PCR measurement of TNF-α and IL-10 in murine BMDMs differentiated towards M1 or M2. Each symbol represents an independent biological sample and the box plot represents the median of the group ± the range. T-test with Mann-Whitney’s *post hoc* test. * = p ≤ 0.05, ** = p ≤ 0.01.
(TIF)

S5 Fig Differential recognition of T3^(-)^ *Y*. *pestis* between human and mice neutrophils. (A) Murine (BMNs) or (B) human (hPMNs) neutrophils were infected with *Y*. *pestis* (Yp) or mutants that either lacked the Yop effectors (T3E) or lacked the Yop effectors and the T3SS [T3(-)] at the indicated MOIs and LTB_4_ was measured 1 h post-infection. Each symbol represents an independent biological infection and the box plot represents the median of the group ± the range. UI = uninfected. One-way ANOVA with Dunnett’s *post hoc* test compared to uninfected. *** = p ≤ 0.001, # = p ≤ 0.0001.
(TIF)

S1 Table Changes in inflammatory lipids during first 48h of pneumonic plague. C57BL/6J mice were infected with 10 x the LD_50_ of *Y*. *pestis* KIM5+ and lungs were harvested at 6, 12, 24, 36, and 48 h post-infection (n = 5). Total lipids were isolated from homogenized lungs and lipids were quantified by LC-MS. Significant changes in lipid concentrations were observed in at least one time point for 63 lipids. Lipids that were below the limit of detection for all time points were excluded from statistical analysis.
(XLSX)

S2 Table Bacterial strains and plasmids used in this study. (DOCX)
