# Supplementary figures and images for: Type 3 secretion system induced leukotriene B4 synthesis by leukocytes is actively inhibited by Yersinia pestis to evade early immune recognition
Source: PLoS Pathog. 2024 Jan 25;20(1):e1011280. doi: 10.1371/journal.ppat.1011280 (PMC10846697; doi:10.1371/journal.ppat.1011280)

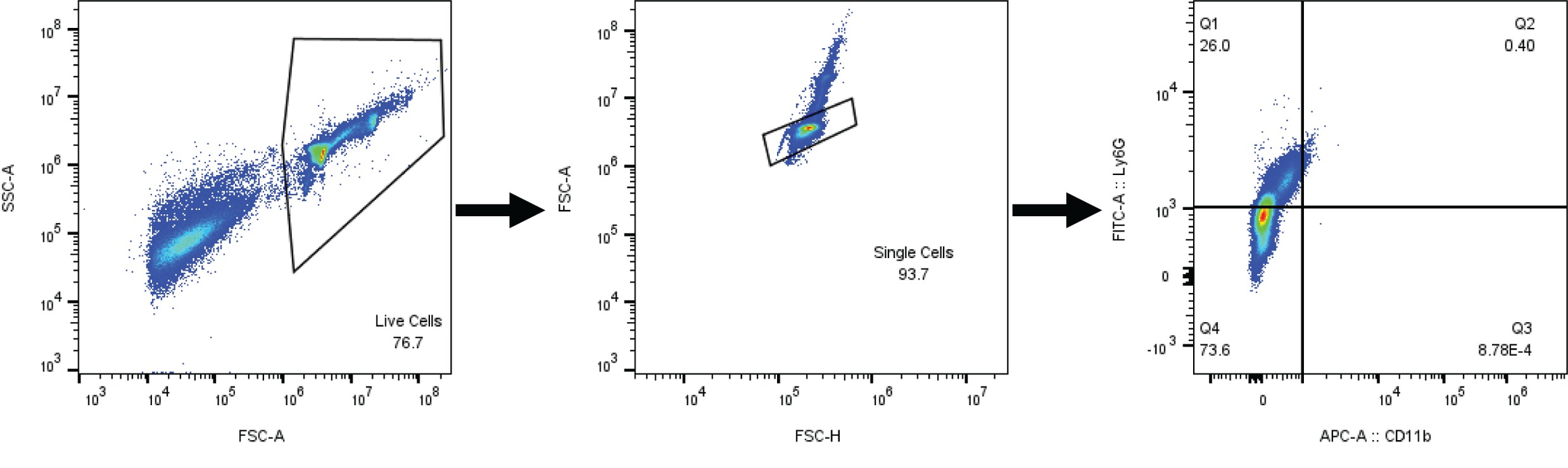

Supplement: S1 Fig — Example gating strategy from the PBS-treated group from Fig 3A. (TIF) [file ppat.1011280.s001.tif]

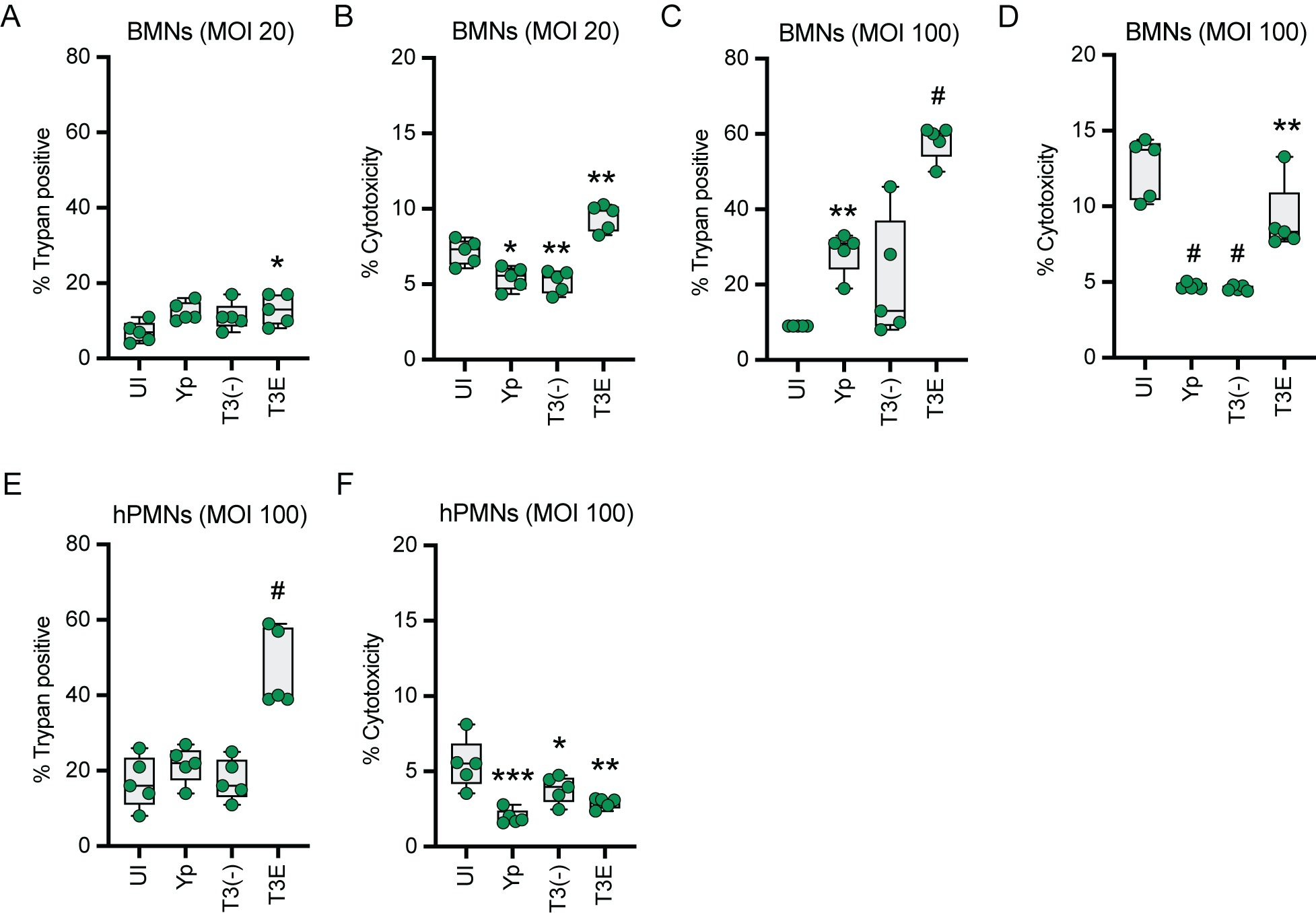

Supplement: S2 Fig — (A-D) Murine (BMNs) or (E-F) human (hPMNs) neutrophils were infected with Y. pestis (Yp) or mutants that either lacked the Yop effectors (T3E) or lacked the Yop effectors and the T3SS [T3(-)] at the indicated MOIs and cell permeability as a function of trypan exclusion or cytotoxicity as a function of LDH release was measured at 1 h post-infection. Each symbol represents an independent biological infection and the box plot represents the median of the group ± the range. UI = uninfected. One-way ANOVA with Dunnett’s post hoc test compared to uninfected. * = p ≤ 0.05, ** = p ≤ 0.01, *** = p ≤ 0.001, # = p ≤ 0.0001. (TIF) [file ppat.1011280.s002.tif]

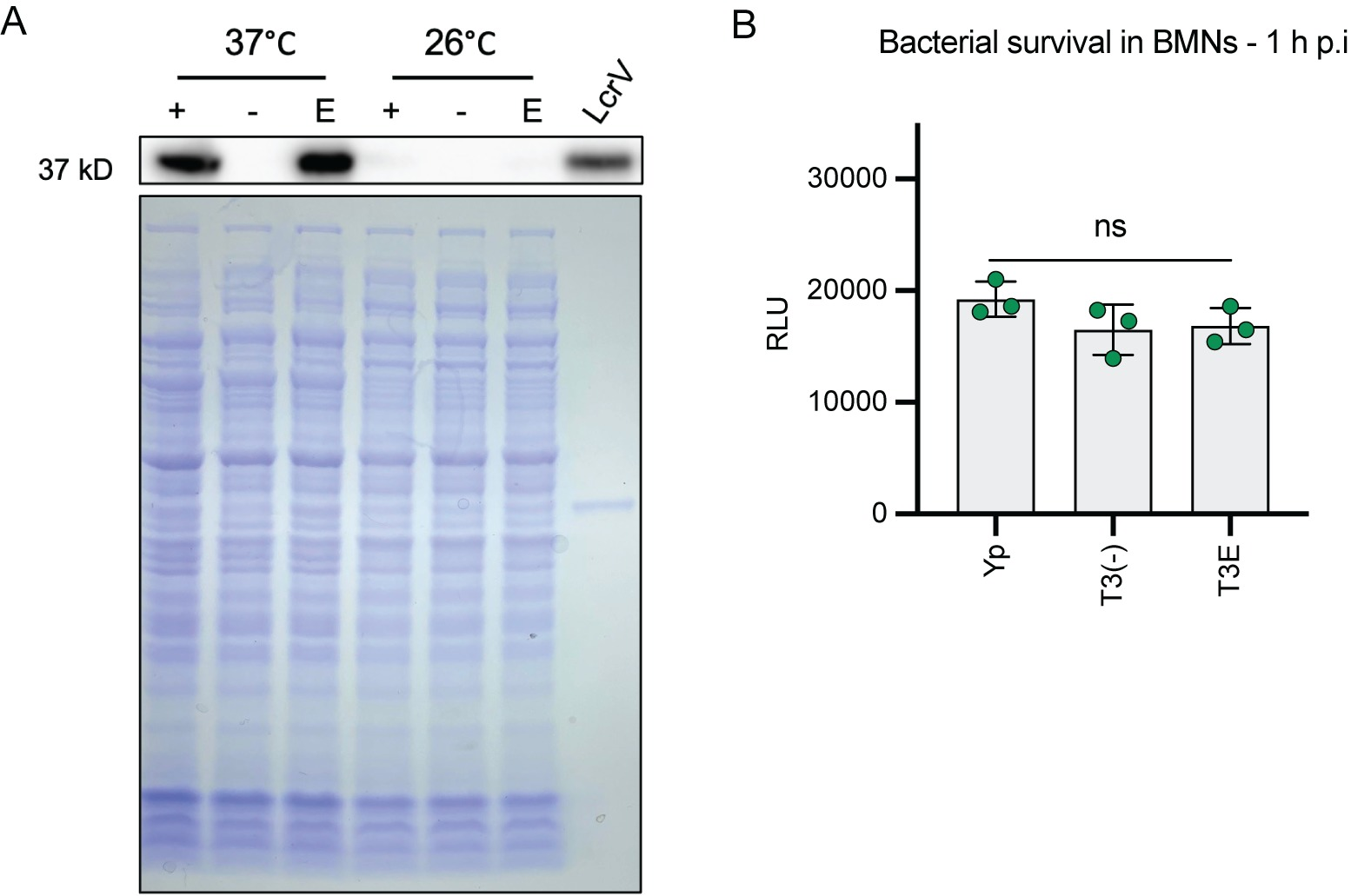

Supplement: S3 Fig — (A) Representative western blot and Coomassie images of Y. pestis lysates (0.1 OD; 1 OD = 3 x 10^8^ CFU) harvested from cultures grown at 37°C or 26°C used for densitometry reported in Fig 6A. (B) Bacterial viability measured by a function of bioluminescence after 1 h infection of neutrophils. + or Yp = Y. pestis,— or T3(-) = Y. pestis T3(-); E or T3E = Y. pestis T3E; LcrV = 0.2 μg recombinant LcrV protein. Each symbol represents an independent biological infection and the bar graph represents the mean ± the standard deviation. One-way ANOVA with Tukey’s post hoc test compared to each condition. ns = not significant. (TIF) [file ppat.1011280.s003.tif]

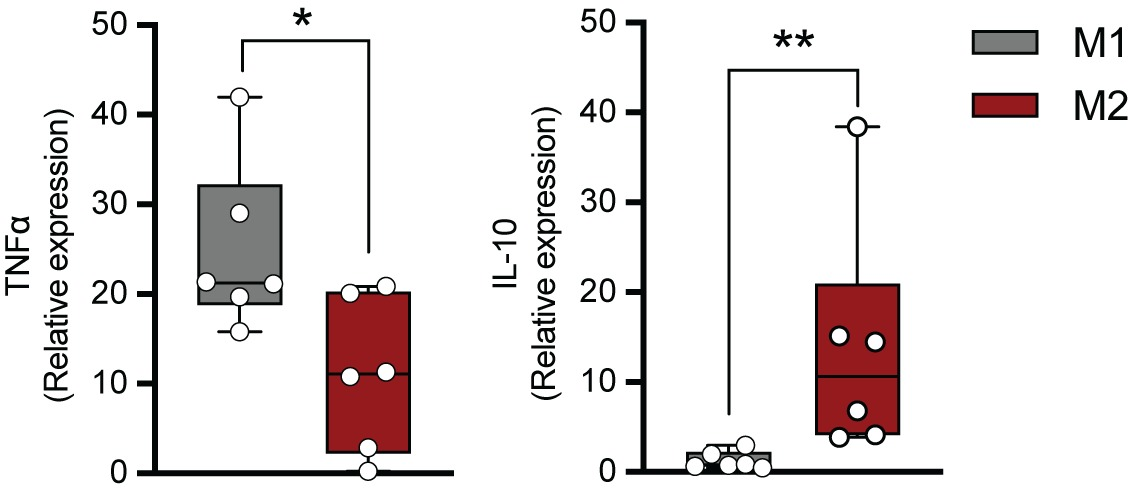

Supplement: S4 Fig — qRT-PCR measurement of TNF-α and IL-10 in murine BMDMs differentiated towards M1 or M2. Each symbol represents an independent biological sample and the box plot represents the median of the group ± the range. T-test with Mann-Whitney’s post hoc test. * = p ≤ 0.05, ** = p ≤ 0.01. (TIF) [file ppat.1011280.s004.tif]

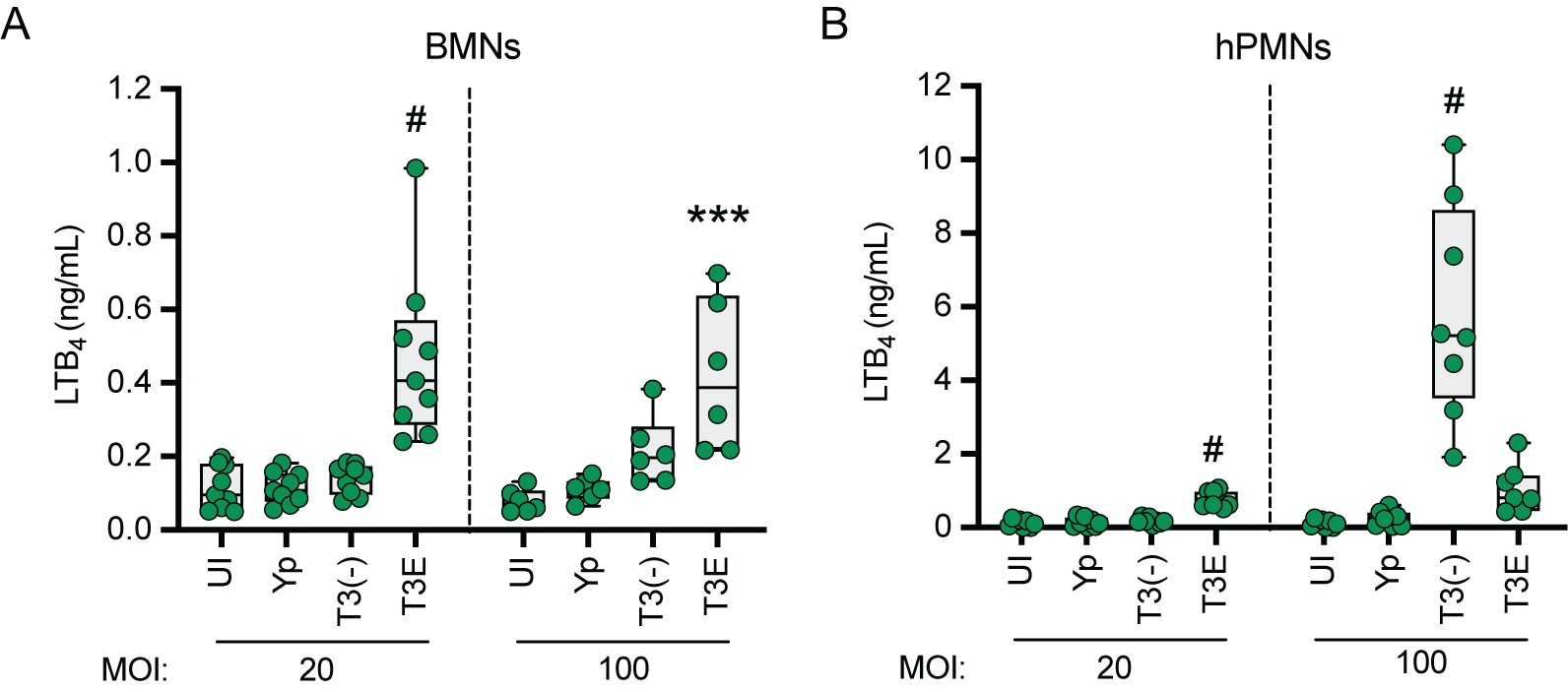

Supplement: S5 Fig — (A) Murine (BMNs) or (B) human (hPMNs) neutrophils were infected with Y. pestis (Yp) or mutants that either lacked the Yop effectors (T3E) or lacked the Yop effectors and the T3SS [T3(-)] at the indicated MOIs and LTB4 was measured 1 h post-infection. Each symbol represents an independent biological infection and the box plot represents the median of the group ± the range. UI = uninfected. One-way ANOVA with Dunnett’s post hoc test compared to uninfected. *** = p ≤ 0.001, # = p ≤ 0.0001. (TIF) [file ppat.1011280.s005.tif]
