# Supplementary material for: Type 3 secretion system induced leukotriene B4 synthesis by leukocytes is actively inhibited by Yersinia pestis to evade early immune recognition
Source: PLoS Pathog. 2024 Jan 25;20(1):e1011280. doi: 10.1371/journal.ppat.1011280 (PMC10846697; doi:10.1371/journal.ppat.1011280)
Supplement: S2 Table — (DOCX) [file ppat.1011280.s007.docx]

| **Descriptive name used in manuscript** | **Genotype** | **Strain ref. #** | **Source** |
| --- | --- | --- | --- |
| **Bacterial Strains** |  |  |  |
| *Y. pestis* KIM5+ | pgm+, pMT1+, pPCP1+, pCD1Ap | X17 | [1] |
| *Y. pestis* CO92 LUX*_pcysZK_* | pgm+, pMT1+, pPCP1+, pCD1+, Lux*_pcysZK_* | MBLYP043 | [2] |
| *Y. pestis* | KIM1001 pgm-, pMT1+, pPCP1+, pCD1+, pML001+ | JG598 | [3] |
| *Y. pestis* T3^(-)^ | KIM1001 pgm-, pMT1+, pPCP1+, pCD1-, pML001+ | JG597 | [3] |
| *Y. pestis* T3E | KIM1001 pgm-, pMT1+, pPCP1+, pCD1+ (yopH^Δ3-467^ yopE^Δ40-197^ yopK^Δ4-181^ yopM^Δ3-408^ ypkA^Δ3-731^ yopJ^Δ4-288^ yopT^Δ3-320^), pML001+ | JG715 | [3] |
| *Y. pestis* T3E +ypkA | KIM1001 pgm-, pMT1+, pPCP1+, pCD1+ (yopH^Δ3-467^ yopE^Δ40-197^ yopK^Δ4-181^ yopM^Δ3-408^ yopJ^Δ4-288^ yopT^Δ3-320^), pML001+ | JG684 | [3] |
| *Y. pestis* T3E +yopE | KIM1001 pgm-, pMT1+, pPCP1+, pCD1+ (yopH^Δ3-467^ yopK^Δ4-181^ yopM^Δ3-408^ ypkA^Δ3-731^ yopJ^Δ4-288^ yopT^Δ3-320^), pML001+ | JG681 | [3] |
| *Y. pestis* T3E +yopH | KIM1001 pgm-, pMT1+, pPCP1+, pCD1+ (yopE^Δ40-197^ yopK^Δ4-181^ yopM^Δ3-408^ ypkA^Δ3-731^ yopJ^Δ4-288^ yopT^Δ3-320^), pML001+ | JG680 | [3] |
| *Y. pestis* T3E +yopJ | KIM1001 pgm-, pMT1+, pPCP1+, pCD1+ (yopH^Δ3-467^ yopE^Δ40-197^ yopK^Δ4-181^ yopM^Δ3-408^ ypkA^Δ3-731^ yopT^Δ3-320^), pML001+ | JG686 | This work |
| *Y. pestis* T3E +yopK | KIM1001 pgm-, pMT1+, pPCP1+, pCD1+ (yopH^Δ3-467^ yopE^Δ40-197^ yopM^Δ3-408^ ypkA^Δ3-731^ yopJ^Δ4-288^ yopT^Δ3-320^), pML001+ | JG682 | [3] |
| *Y. pestis* T3E +yopM | KIM1001 pgm-, pMT1+, pPCP1+, pCD1+ (yopH^Δ3-467^ yopE^Δ40-197^ yopK^Δ4-181^ ypkA^Δ3-731^ yopJ^Δ4-288^ yopT^Δ3-320^), pML001+ | JG683 | [3] |
| *Y. pestis* T3E +yopT | KIM1001 pgm-, pMT1+, pPCP1+, pCD1+ (yopH^Δ3-467^ yopE^Δ40-197^ yopK^Δ4-181^ yopM^Δ3-408^ ypkA^Δ3-731^ yopJ^Δ4-288^), pML001+ | JG685 | [3] |
| *Y. pestis* T3E *yopB* | KIM1001 pgm-, pMT1+, pPCP1+, pCD1+ (yopH^Δ3-467^ yopE^Δ40-197^ yopK^Δ4-181^ yopM^Δ3-408^ ypkA^Δ3-731^ yopJ^Δ4-288^ yopT^Δ3-320^yopB ^Δ7-396^), pML001+ | YPA322 | This work |
| *Y. pestis* *yopB*::c*yopB* | KIM1001 pgm-, pMT1+, pPCP1+, pCD1+ (yopH^Δ3-467^ yopE^Δ40-197^ yopK^Δ4-181^ yopM^Δ3-408^ ypkA^Δ3-731^ yopJ^Δ4-288^ yopT^Δ3-320^), pML001+ | YPA362 | This work |
| *E. coli* | *E. coli* DH5α pGEN222::mCherry | LOU123 | This work |
| *Salmonella* *enterica* Typhimurium | *S*. *enterica* Typhimurium LT2 pGENLux | LOU120  ATCC 14028s | This work |
| *Klebsiella pneumoniae* *manC* | KPPR1S *ΔmanC* | LOU171 | [4] |
| **Plasmids** |  |  |  |
| pML001 | Luciferase bioreporter | NA | [3] |
| pGENlux | Luciferase bioreporter | MBL343 | [5] |

**S2 Table. Bacterial strains and plasmids used in this study.**

**References for supporting information**

1. Gong S, Bearden SW, Geoffroy VA, Fetherston JD, Perry RD. Characterization of the *Yersinia pestis* Yfu ABC inorganic iron transport system. Infect Immun. 2001;69(5):2829-37. Epub 2001/04/09. doi: 10.1128/IAI.67.5.2829-2837.2001. PubMed PMID: 11292695; PubMed Central PMCID: PMCPMC98231.

2. Pulsifer AR, Vashishta A, Reeves SA, Wolfe JK, Palace SG, Prouix MK, et al. Redundant and cooperative roles for *Yersinia pestis* yop effectors in the inhibition of human neutrophil exocytic responses revealed by gain-of-function approach. Infect Immun. 2020;88(3):1-16. doi: 10.1128/IAI.00909-19.

3. Palace SG, Proulx MK, Szabady RL, Goguen JD. Gain-of-function analysis reveals important virulence roles for the *Yersinia pestis* type III secretion system effectors YopJ, YopT, and YpkA. Infect Immun. 2018;86(9):1-11. doi: 10.1128/IAI.

4. Fodah RA, Scott JB, Tam HH, Yan P, Pfeffer TL, Bundschuh R, et al. Correlation of Klebsiella pneumoniae comparative genetic analyses with virulence profiles in a murine respiratory disease model. PLoS ONE. 2014;9(9):e107394. Epub 20140909. doi: 10.1371/journal.pone.0107394. PubMed PMID: 25203254; PubMed Central PMCID: PMCPMC4159340.

5. Lane MC, Alteri CJ, Smith SN, Mobley HL. Expression of flagella is coincident with uropathogenic *Escherichia coli* ascension to the upper urinary tract. Proc Natl Acad Sci U S A. 2007;104(42):16669-74. Epub 20071009. doi: 10.1073/pnas.0607898104. PubMed PMID: 17925449; PubMed Central PMCID: PMCPMC2034267.
